# Integrin α_D_β_2_ (CD11d/CD18) Is Expressed by Human Circulating and Tissue Myeloid Leukocytes and Mediates Inflammatory Signaling

**DOI:** 10.1371/journal.pone.0112770

**Published:** 2014-11-21

**Authors:** Yasunari Miyazaki, Adriana Vieira-de-Abreu, Estelle S. Harris, Amrapali M. Shah, Andrew S. Weyrich, Hugo C. Castro-Faria-Neto, Guy A. Zimmerman

**Affiliations:** 1 Department of Respiratory Medicine, Tokyo Medical and Dental University, 1-5-45 Yushima, Bunkyo-Ku, Tokyo, 113-8519, Japan; 2 Department of Internal Medicine, University of Utah School of Medicine, Salt Lake City, Utah 84112, United States of America; 3 Program in Molecular Medicine, University of Utah School of Medicine, Salt Lake City, Utah 84112, United States of America; 4 Laboratório de Immunofarmacologia, Instituto Oswaldo Cruz, Fundacão Oswaldo Cruz, Rio de Janeiro, Brazil; National Institute of Environmental Health Sciences, United States of America

## Abstract

Integrin α_D_β_2_ is the most recently identified member of the leukocyte, or β_2_, subfamily of integrin heterodimers. Its distribution and functions on human leukocytes have not been clearly defined and are controversial. We examined these issues and found that α_D_β_2_ is prominently expressed by leukocytes in whole blood from healthy human subjects, including most polymorphonuclear leukocytes and monocytes. We also found that α_D_β_2_ is displayed by leukocytes in the alveoli of uninjured and inflamed human lungs and by human monocyte-derived macrophages and dendritic cells, indicating broad myeloid expression. Using freshly-isolated human monocytes, we found that α_D_β_2_ delivers outside-in signals to pathways that regulate cell spreading and gene expression. Screening expression analysis followed by validation of candidate transcripts demonstrated that engagement of α_D_β_2_ induces mRNAs encoding inflammatory chemokines and cytokines and secretion of their protein products. Thus, α_D_β_2_ is a major member of the integrin repertoire of both circulating and tissue myeloid leukocytes in humans. Its broad expression and capacity for outside-in signaling indicate that it is likely to have important functions in clinical syndromes of infection, inflammation, and tissue injury.

## Introduction

Integrins are plasma membrane αβ heterodimers that are broadly distributed on metazoan cells and that mediate critical functions including adhesion, homing, mechanical linkage of cytoskeletal elements to extracellular matrix, cell-cell interactions, “outside-in” signaling, and cell survival [1]–[3]. A subgroup of integrins defined by presence of the β_2_ integrin subunit in non-covalent linkage with specific α subunits has restricted expression on leukocytes, and is variably termed the leukocyte integrin subfamily, the β_2_ integrin subfamily, the CD18 integrins, or the leukointegrins [1],[4]. The leukocyte integrins have essential activities in physiologic and pathologic inflammatory and immune responses. Their requirement for host defense against invading pathogens is clearly demonstrated by heritable leukocyte adhesion deficiency syndromes in humans in which β_2_ integrin expression is dramatically reduced or absent, or intracellular mechanisms that control the adhesive functions of these integrins are disrupted [4]–[8]. Similarly, targeted deletion of β_2_ integrins, or key intracellular factors that regulate their activation, leads to defects in host defense and inflammation in murine models [3],[8]–[11].

The leukocyte integrins include four heterodimers: α_L_β_2_ (CD11a/Cd18; LFA-1), α_M_β_2_ (CD11b/CD18, MAC-1), α_X_β_2_ (CD11c/CD18), and α_D_β_2_ (CD11d/CD18) [1]–[5],[8]. As with other members of the broad integrin family, under physiologic conditions the α subunits are not expressed on the cell surface in the absence of linkage to the β partner, and vice versa [3]. Integrin α_D_β_2_ is the most recently identified leukocyte integrin family member. In contrast to the other three β_2_ integrins, little is known about it [8]. When the α_D_ subunit was originally cloned, molecular characterization suggested that the α_D_β_2_ heterodimer may have novel modes of expression and regulation, and a unique pattern of ligands [12]. More recently, genetic deletion of α_D_ in mice indicated that α_D_β_2_ has complicated activities in thymocyte function, T cell development, and superantigen stimulation [13], and that it mediates adhesion and function of specific classes of macrophages [14]. Consistent with this, the α_D_ mRNA transcript, designated *ITGAD* in humans and *itgad* in mice [15],[16], is enriched in some subclasses of murine macrophages [17]. Furthermore, disease models demonstrate that α_D_β_2_ influences systemic inflammatory responses and survival in experimental models of malaria and of *Salmonella* infection [14] (Nascimento DO, Vieira-de-Abreu A, et al., manuscript submitted). In addition, administration of monoclonal antibodies against α_D_ improved neurologic outcomes after spinal cord or traumatic brain injury in rats, presumably by reducing accumulation of neutrophils and macrophages in injured nervous tissues and thereby blunting inflammatory damage [18]–[20]. Thus, the studies to date indicate that α_D_β_2_ has key functional activities in inflammation, responses to pathogens, and tissue injury in experimental animals. In parallel, human α_D_β_2_ was recently proposed to mediate signaling between polymorphonuclear leukocytes (PMNs, neutrophils) and natural killer cells in complex interactions with a dendritic cell subclass [21],[22]. Nevertheless, assignment of inflammatory activities to α_D_β_2_ is limited by controversy regarding its expression by human leukocytes and gaps in knowledge regarding its functions. Published reports and reviews state that α_D_β_2_ is not displayed by circulating human leukocytes, conclude that it is primarily expressed on eosinophils, or state that it is largely expressed by macrophages in atherosclerosis and in other inflammatory syndromes [3],[23]–[25]. In addition, signaling activities of α_D_β_2_ on human leukocytes are unexplored. In this study we examined these issues, focusing on myeloid leukocytes from human blood.

## Materials and Methods

### Cells and tissues

Human leukocytes were examined in whole blood samples from healthy subjects or were isolated from the blood of healthy volunteers. Procedures for collecting blood samples from normal subjects and for examination of autopsy samples by microscopy and immunocytochemistry (see below) were approved by the University of Utah Institutional Review Board. For studies of monocytes, macrophages, and dendritic cells, monocytes were isolated by countercurrent elutriation [26] or by selection using microbeads (Miltenyi Biotech) with modifications of methods that we have previously described for other human leukocytes [27]. For microbead separations of monocytes, the mononuclear layer (MNL) of cells was first separated from anticoagulated (ACD or EDTA) whole blood and then incubated with a blocking anti-Fc receptor mAb (Stem Cell Technologies). Granulocytes and NK cells were removed by incubation of the cell suspension with microbeads coated with anti-CD15 and anti-CD56 using the Miltenyi Magnetic Sorter. The suspension was then incubated with anti-CD14-coated microbeads and unfractionated monocytes were isolated by magnetic sorting. To isolate monocytes subpopulations, the mononuclear suspension was pre-incubated with the anti-Fc blocking mAb and immunodepleted of granulocytes and NK cells as outlined above. The cell suspension was then incubated with anti-CD16-coated microbeads and the CD16^+^ subset of monocytes was positively selected by magnetic sorting. The remaining cell suspension was then incubated with anti-CD14-coated beads and the CD16^−^ subset of monocytes was further isolated by magnetic separation. Isolated unfractionated monocytes and monocytes subsets were suspended in Hanks Balanced Salt Solution (HBSS) with 0.5% albumin (HBSS/A) and used in acute experiments or cultured under specific conditions to induce their differentiation to monocyte-derived macrophages (MDM) or monocyte-derived dendritic (MDD) cells [28],[29]. Staining of α_D_β_2_ on leukocytes in mouse blood and tissues was done as described [14] after approval of the protocols by the Animal Welfare Committee of the Oswaldo Cruz Foundation and the University of Utah Institutional Animal Care and Use Committee.

### Antibodies and control proteins

Monoclonal antibodies (mAb) with reactivity against α_D_ were developed and characterized as described [12],[30],[31] and were generously provided by investigators at ICOS corporation. Monoclonal antibodies against α_M_, α_L_, and α_X_ were purchased (Serotec, Dako). Additional monoclonal antibodies used for flow cytometry are mentioned below. Non-immune murine IgG1 and human serum albumin were from R&D Systems and Baxter Healthcare, respectively.

### Whole blood flow cytometry and flow cytometry of isolated monocytes and monocyte subpopulations

Flow cytometry was done using modifications of previously-described procedures [32]. Following informed consent, whole blood was drawn into 8.6 mL sterile acid-citrate-dextrose (ACD; 1.4 mL ACD/8.6 mL blood) vacutainer tubes (Becton Dickinson, Franklin Lakes, NJ, USA; abbreviated BD in this section), inverted to insure adequate mixing, and transported at room air temperature (20–25°C) to the laboratory within 30 minutes. Whole blood samples were mixed with FACs lysis buffer (BD; diluted into HBSS with 0.5% human serum albumin and 0.1% sodium azide) containing directly-conjugated monoclonal antibodies (see below) and kept at 4°C until analyzed.

**Figure 1:**
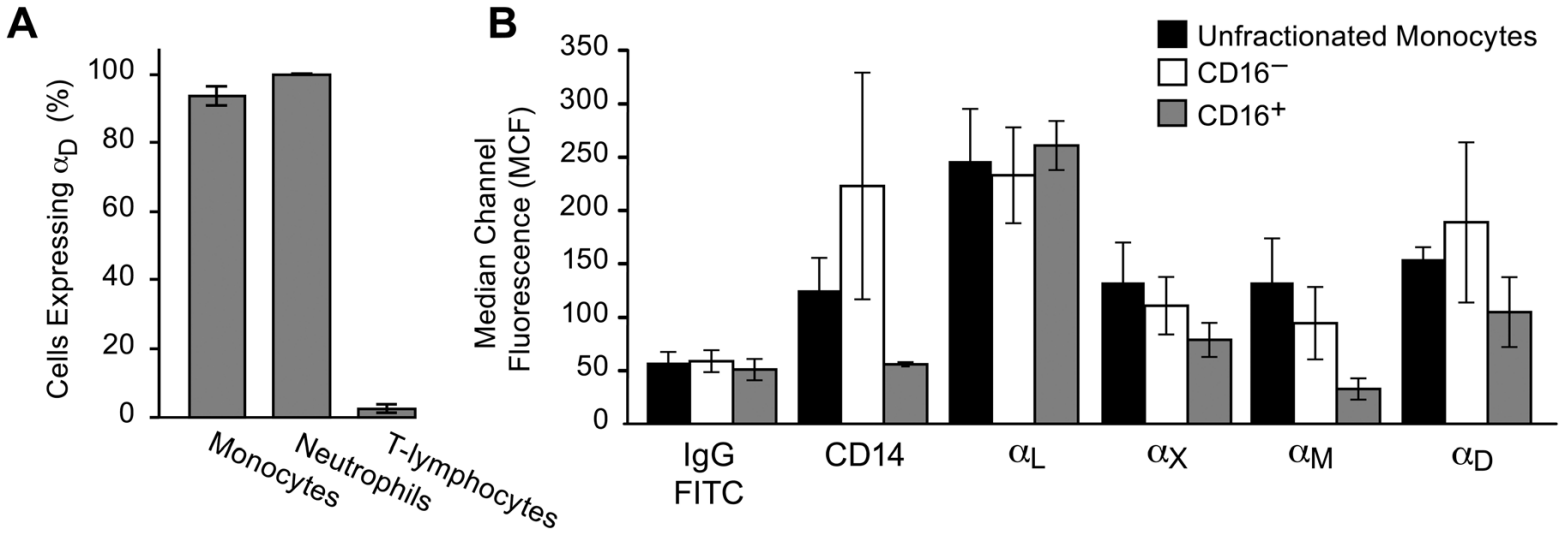
Integrin α_D_β_2_ is highly expressed on myeloid leukocytes in human blood. A. Whole venous blood from healthy volunteers was fixed and expression of α_D_β_2_ on leukocyte subtypes was examined using mAb 169A (anti-α_D_) and FITC-conjugated mAbs against CD14, CD15, and CD3 as described in Materials and Methods. The percent of each cell type positive for α_D_β_2_ is indicated by the bars. The error bars indicate the mean and SD of determinations using samples from four subjects. B. Monocytes were separated from venous blood of healthy volunteers and further separated into CD16^+^ and CD16^−^ CD14^+^ subpopulations as described in Materials and Methods. Surface expression of α_D_β_2_, α_L_β_2_, α_M_β_2_, and α_X_β_2_ was examined by flow cytometry using FITC- or ALEXA-488-conjugated antibodies and isotype-matched IgG controls. Cells in each monocyte fraction were also stained for CD14. These data indicate means and standard deviations in results from 3 experiments using samples from different subjects.

Flow cytometry was performed using a FACScan Analyzer (BD Biosciences, San Jose, CA, USA) with software for analysis (BD) immediately or within 24 hours of fixation. The flow cytometer was calibrated daily and cleaned carefully before each sample acquisition. Commercial FITC-conjugated monoclonal antibodies against CD14, CD16, CD15, and CD3 and IgG controls were obtained from BD Biosciences. Monoclonal antibody 169A, against α_D_ [12], was directly conjugated to alexa 647 (Molecular Probes labeling kit) or alexa 488 (Intvitrogen labeling kit). Leukocyte subsets were selected by gating on white blood cells (excluding platelets) using side and forward scatter profiles (25,000 events) and then selecting positive events using a two-parameter dot plot displaying FL1 (FITC-labeled antibodies against specific markers as indicated above) and FL4 (anti-α_D_) to quantify the percentage of cells expressing α_D_. An example is shown in Figure S1. The monocyte population was identified by gating on CD14/FL1-positive cells that had a characteristic side scatter profile. The neutrophil population was identified by gating on CD15/FL1-positive cells with a characteristic neutrophil side scatter profile, and T lymphocytes were identified by gating on CD3/FL-1-positive cells with an appropriate side scatter profile (Figure S1). Expression of α_D_β_2_ by each leukocyte subpopulation was determined by measuring the FL4 intensity of staining with the fluorescent anti-α_D_ mAb (Figure S1; Figure 1A).

In additional experiments we also analyzed isolated unfractionated monocytes and microbead-separated CD16-positive and CD16-negative monocyte subpopulations (see above) by flow cytometry using CD14, α_D_, α_M_, α_L_, and α_X_ as the surface markers and approaches as outlined above. Monoclonal antibodies against α_M_, α_L_, and α_X_ were obtained from Serotec and conjugated with alexa 488 (Intvitrogen labeling kit). Isotype-matched non-immune IgG was used as a negative control. A summary of these studies is shown in Figure 1B.

### Immunocytochemistry and immunohistochemistry

**Figure 2:**
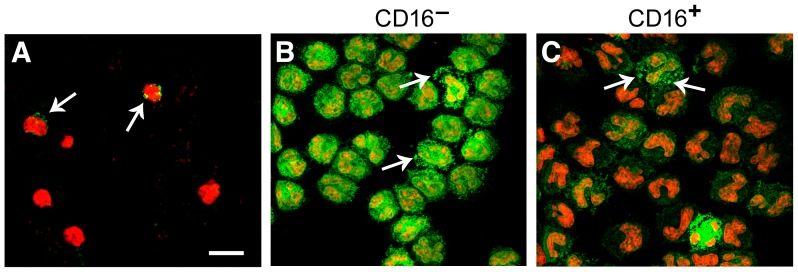
Integrin α_D_β_2_ is expressed by inflamed murine leukocytes and by unstimulated circulating human monocytes. A. Leukocytes in whole cardiac blood from a mouse infected with the rodent malarial parasite *Plasmodium berghei* ANKA [14] were stained for α_D_β_2_ (arrows). B, C. Monocytes were first separated from an unfractionated mononuclear cell suspension from the peripheral blood of a healthy human volunteer and then further separated into CD16^+^ and CD16^−^ subpopulations as described in Materials and Methods. The CD16^−^ CD14^+^ (B) and CD16^+^ (C) monocyte preparations were then fixed, permeabilized, and stained for α_D_ (green fluorescence) using anti-α_D_ mAb 169A. Propidium iodide was used to identify nuclei. In additional experiments isotype-matched non-immune IgG was used as the first immunoglobulin in the staining procedure to control for mAb 169A (Figure S2). In both monocyte subsets, α_D_β_2_ staining had a granular pattern and in some areas there were large clusters of the integrin that appeared to be on or near the surface (arrows). An additional experiment indicated that α_D_β_2_ also clusters on human neutrophils (Figure S3).

Staining of α_D_β_2_ on monocytes, neutrophils, monocyte-derived macrophages, and monocyte-derived dendritic cells was done as previously described [33]. Briefly, the cells were incubated with anti-α_D_ mAb or with non-immune IgG_1_ followed by washing and incubation with FITC-conjugated goat anti-mouse IgG (Molecular Probes). After additional washes, the cells were examined by confocal microscopy (Figure 2 and Figure S2 and S3). Immunohistochemical analysis of fixed samples of lung from individuals who died without evidence of lung injury or inflammation, or with acute lung injury or the acute respiratory distress syndrome, was accomplished as described [34].

### Assays of chemokine and cytokine release

Interleukin 8 (IL-8), interleukin 1 (IL-1), and monocyte chemotactic protein 1 (MCP-1) were measured by ELISA using commercially-available antibodies (Duoset, R&D Systems) as previously reported [35],[36]. There was substantial donor-to-donor variability in maximal chemokine and cytokine release, as has been previously reported for activated human monocytes (see “Results”). Supporting tables display raw data from multiple experiments in which cytokine and chemokine measurements were made at an 8 hr time point to illustrate this point.

### Assays of spreading and altered gene expression by isolated human monocytes

Monocyte spreading and altered expression of inflammatory genes in response to engagement of α_D_β_2_ or other leukocyte integrins by immobilized mAb were examined using previously-published approaches [32],[37]. For assays of altered gene expression, we first examined mRNA transcript profiles using microarray analysis as previously described in detail [35],[37]. Validation of these preliminary results by reverse transcriptase polymerase chain reaction (RT PCR) was then done using methods as described [38],[39].

### Monocyte incubations with purified intercellular adhesion molecule 3 (ICAM-3)

Culture wells in 4 well plates (NUNC) were coated with recombinant ICAM-3 (R&D Systems) (10 µg/ml) or human serum albumin (HSA) (2%) for 1 hr at 37°C. The wells were washed three times with HBSS, blocked with human serum albumin (2%) for 3 hr. at room temperature, washed with Tween-20 (0.1%) in HBSS, and washed again twice with HBSS. Isolated monocytes (2×10^6^) were pre-incubated in medium 199 (M199) alone or in M199 with anti-α_D_ mAbs 169A (non-blocking) or 204I (blocking) (10 µg/ml) for 60 min at 4°C and then incubated in ICAM-3-coated wells. Polymyxin B (10 µg/ml) was added to the monocyte suspensions to prevent spurious activation of the leukocytes by contaminating lipopolysaccharide. After 18 hr. incubations of monocytes on immobilized ICAM-3 or in albumin-coated wells the supernatants were removed, centrifuged, and frozen at −80°C until analysis for IL-8 by ELISA. Statistical analysis was done using Tukey's multiple comparison and Newman-Keuls multiple comparison tests.

## Results

### Integrin α_D_β_2_ is expressed on multiple classes of circulating human leukocytes, and is prominently displayed by myeloid leukocytes

Flow cytometric analysis of fixed whole venous blood and immunocytochemical analysis of isolated cells from healthy volunteers revealed that the majority of circulating human neutrophils and monocytes express α_D_β_2_ (Figures 1A; 2B, C). A much smaller fraction of circulating T-lymphocytes was positive when whole blood from healthy subjects was examined (Figure 1A). In contrast, previously-published observations indicate that α_D_β_2_ is expressed on a small fraction of circulating leukocytes (<1% under basal conditions) in the blood of mice of several genetic backgrounds [13],[14], although it may be induced on circulating murine leukocytes in response to infection and inflammation (Figure 2A and ongoing studies). These findings demonstrate that α_D_β_2_ is robustly expressed by human leukocyte subsets and, together with earlier studies [13],[14], that the pattern of expression of α_D_β_2_ on circulating leukocytes under basal conditions varies substantially between mice and humans.

We next examined the pattern of expression of α_D_β_2_ on human monocytes, since these leukocytes are critical immune effector cells and are precursors of macrophages and dendritic cells [40],[41]. In addition, some circulating α_D_^+^ leukocytes in the blood of mice have morphologic features suggestive of monocytes, including high nuclear-to-cytoplasmic ratios and indented nuclei (Figure 2A), and α_D_β_2_ is reported to be induced on circulating murine monocytes in response to sterile inflammation [25]. We found that integrin α_D_β_2_ is highly expressed on unfractionated human monocytes when examined by flow cytometry after separation from other blood cells (Figure 1B). After further separation of monocyte subtypes (Figures 1B; 2B, C), mean surface expression of α_D_β_2_ was higher on the CD16 negative (CD16^−^) monocyte subpopulation compared to CD16 positive (CD16^+^) monocytes (P = 0.0435), as was mean surface expression of α_X_β_2_ (P = 0.0078) (Figure 1B). In both CD16^−^ and CD16^+^ monocyte subpopulations the expression of α_D_β_2_ on resting, unstimulated cells was similar to, or greater than, that of α_M_β_2_ and α_X_β_2_, although less than expression of α_L_β_2_. Together, the observations in Figures 1 and 2 indicate that α_D_β_2_ is a major integrin on circulating human monocytes, although there is an element of variation among subtypes.

### Integrin α_D_β_2_ is expressed by human macrophages *in vivo* and by monocyte-derived macrophages and dendritic cells during differentiation *in vitro*

**Figure 3:**
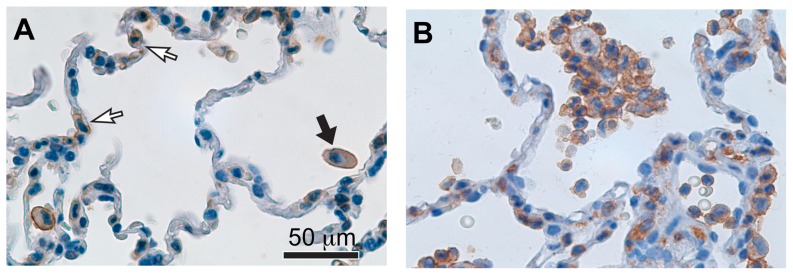
Integrin α_D_β_2_ is expressed by leukocytes in human lung. A. Tissue samples from a human subject with normal lungs who underwent autopsy after fatal head injury were fixed and stained for α_D_ with mAb 169A as outlined in Materials and Methods and [34]. Microscopic evaluation revealed α_D_β_2_^+^ macrophages in alveolar spaces (black arrow). There are also scattered α_D_^+^ cells in alveolar walls (white arrows), which may be interstitial macrophages and/or dendritic cells. This image is representative of findings from analysis of lung tissue from three subjects who died without lung disease or injury. B. Autopsy samples from a patient who died with acute respiratory distress syndrome [34], [45] were fixed, stained for α_D_, and examined by light microscopy. Numerous α_D_β_2_^+^ macrophages were detected in alveolar spaces and walls. In some fields α_D_β_2_^+^ neutrophils were also present (not shown). Lung samples from 3 patients who died with acute lung injury or ARDS as defined by consensus criteria [44] were examined. We found α_D_β_2_^+^ leukocytes in the alveoli in each case.

**Figure 4:**
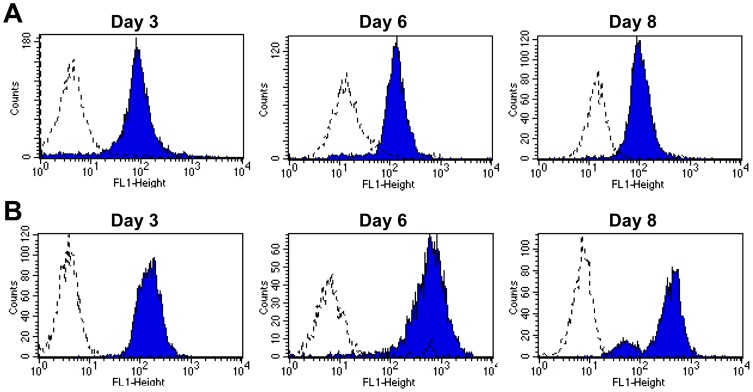
Human monocyte-derived macrophages and dendritic cells express integrin α_D_β_2_ during differentiation in culture. Unfractionated monocytes were separated from the venous blood of healthy subjects by positive selection and differentiated to (A) macrophages or (B) monocyte-derived dendritic cells using procedures and protocols outlined in Materials and Methods. MDM and MDDC were examined for expression of α_D_β_2_ by flow cytometry on days 3, 6, and 8 in culture. These results are representative of findings in three experiments using cells from different subjects.

In previous studies little or no α_D_β_2_ expression was detected in rodent lungs under basal conditions [14],[42] or on alveolar macrophages from dogs [43]. In contrast, we observed frequent α_D_-positive leukocytes in autopsy samples of lungs from patients without evidence of pulmonary disease or injury at the time of death, including leukocytes in the alveolar wall and cells with morphology of alveolar macrophages in the alveolar space (Figure 3A and data not shown). In addition, we detected abundant α_D_β_2_-positive leukocytes in alveoli in autopsy samples from patients with acute lung injury (ALI) or its most severe form, acute respiratory distress syndrome (ARDS) [44] (Figure 3B and data not shown). ALI and ARDS were diagnosed according to consensus criteria established in the field [44]. Macrophages and dendritic cells, in addition to emigrating monocytes and PMNs, are found in variable numbers in the alveolar spaces and walls in ALI and ARDS [45]. Consistent with tissue analysis (Figure 3), we found that α_D_β_2_ is continuously expressed when unfractionated human monocytes are differentiated to macrophages in vitro (Figure 4A; Table 1). It is also expressed at high level by immature myeloid dendritic cells differentiated from human monocytes (Figure 4B; Table 1). Thus, α_D_β_2_ is robustly displayed by human myeloid leukocytes in tissue compartments under basal and inflammatory conditions (Figure 3), by model macrophages and dendritic cells (Figure 4; Table 1), and by circulating human myeloid leukocytes (Figures 1 and 2).

**Table 1:** Integrin α_D_β_2_ is Dynamically Expressed by Human Monocyte-derived Macrophages (MDM) and Dendritic Cells (MDDC).

Experiment 1: MDM	
Agonist	α_D_ (MFI; % positive cells indicated in parentheses)
Control	88 (90)
LPS	40 (95)
IL-4	97 (87)
IFNg	83 (84)

Experiment 2: MDM	
Agonist	α_D_
Control	55 (88)
LPS	30 (97)
IL-4	75 (99)
IFNg	60 (99)

Experiment 3: MDDC	
Agonist	α_D_
Control	28 (89)
LPS	11 (54)

Isolated human monocytes were cultured in human serum (Experiment 1) or M-CSF (Experiment 2) for 3 days to induce differentiation into monocyte-derived macrophages [28],[29]. After 6 days in culture they were treated with LPS, with IFNg as a stimulus for “classic activation”, or with IL-4 as a stimulus for “alternative activation” [46],[47]. In a third experiment monocytes were cultured in GM-CSF and IL-4 to induce differentiation to immature MDDC (“control”); they were then treated with LPS to trigger transition to mature MDDC (“LPS”) [29]. Results are expressed as mean fluorescence intensity (MFI), with the percentage of positive cells in parentheses.

Monocyte-derived macrophages and dendritic cells (MDDC) alter their phenotypes and functions in response to bacterial products, cytokines, and agonists that induce differentiation [40], but it is unknown if α_D_β_2_ expression is altered under these conditions. In two experiments in which monocytes were cultured using different stimuli for differentiation – serum, or macrophage colony stimulating factor (M-CSF) [28],[29] – most monocyte-derived macrophages expressed α_D_β_2_ (mean 89%, experiments 1 and 2 in Table 1). Treatment with lipopolysaccharide (LPS) further increased the number of MDM expressing α_D_β_2_ (mean 96%), although the mean fluorescence intensity (MFI) decreased, suggesting that the number of cells that express α_D_β_2_ and the magnitude of its surface expression can vary independently. Expression of α_D_β_2_ was sustained when MDM were incubated with interferon gamma (IFNγ) or interleukin 4 (IL-4) to induce polarization to “classic” or “alternatively-activated” functional phenotypes [46],[47] (Table 1). In a third experiment, monocytes were first induced to differentiate to immature MDDC, and then treated with LPS to induce differentiation to mature MDDC [29]. The majority of immature MDDC (89%) expressed α_D_β_2_; the number of positive cells decreased with a parallel reduction in MFI in response to stimulation with LPS (Figure 4; Experiment 3 in Table 1). These results indicate that α_D_β_2_ is expressed by MDM and MDDC generated in response to varying conditions for differentiation, and when these immune effector cells are stimulated by agonists that induce different functional phenotypes. Further, the expression of α_D_β_2_ on human MDM and MDDC may be dynamically altered in response to inflammatory mediators, although the majority of these leukocytes express it under basal conditions in vitro (Table 1).

### Engagement of integrin α_D_β_2_ on human monocytes transmits outside-in signals and induces new gene expression

Integrin α_D_β_2_ mediates adhesion of murine and human leukocytes and cell lines in vitro [14],[24],[25],[30], consistent with adhesive activities of β_2_ integrins that contribute to leukocyte targeting and localization [1]–[5],[8]. Integrins, including leukocyte integrins, also transmit “outside-in” signals that are linked to key intracellular transduction cascades [1],[4],[48],[49]. These pathways influence functional responses, including transcriptional and post-transcriptional gene expression. Nevertheless, outside-in signaling by α_D_β_2_ has not previously been examined; furthermore the putative cytoplasmic sequence of α_D_ differs substantially from that of the other leukocyte integrin α subunits (10–18% identity), suggesting that α_D_β_2_ may not share outside-in signaling pathways with α_L_β_2_, α_m_β_2_, and α_x_β_2_ [12]. Therefore, we asked if α_D_β_2_ transmits outside-in signals using freshly-isolated human monocytes as the model.

We first examined monocyte spreading on immobilized monoclonal antibodies raised against α_D_β_2_ as a screening assay to determine if engagement of this integrin delivers signals that alter functional responses. This strategy has previously been employed to ask if engagement of other adhesion molecules on myeloid leukocytes mediates specific outside-in signaling [32],[50]–[53]. Wells coated with non-immune IgG or albumin served as controls. In two experiments, monocytes actively spread on immobilized anti-α_D_ mAbs 169B and 217I (coating concentration 10 µg/ml) to a much greater extent than on immobilized isotype-matched IgG or HSA over a 30 min – 8 hr incubation period, with maximal spreading at 2 hr–8 hr (Table S1). Four other immobilized anti- α_D_ mAb induced lesser degrees of spreading that was not consistently different from control in parallel incubations under the same conditions. In each assay, spreading of monocytes on an immobilized mAb against α_M_ was also examined in parallel as a comparative assay for outside-in signaling triggered by engagement of α_M_β_2_ integrin; 60 to 90% of the leukocytes spread on immobilized anti- α_M_ at 2–8 hr in the two experiments. The data from these experiments are tabulated in detail in Table S1. The incubations utilizing immobilized anti-α_D_ mAbs yielded preliminary evidence that α_D_β_2_ on human monocytes transmits outside-in signals, and also provided additional evidence that α_D_β_2_ is expressed on circulating human monocytes that complemented analysis by immunostaining and flow cytometry (Figures 1,2). In subsequent experiments examining outside-in signaling by α_D_β_2_ we focused on mAb 169B and 217I as activating antibodies because they were the most potent of the anti-α_D_ mAb in the spreading assays (Table S1).

We used a similar approach to determine if engagement of α_D_β_2_ alters the pattern of expressed genes in monocytes. Isolated monocytes were incubated on immobilized anti-α_D_ mAb 169B, or on wells coated with isotype-matched control IgG, and mRNA was extracted and interrogated by microarray analysis. In this screening experiment transcripts encoding a variety of factors were increased or decreased by engagement of α_D_β_2_ (Table S2). We chose two candidate mRNAs – *estrogen receptor α* (*ERα*) and *interleukin 8* (*IL-8*) – for validation. They were selected because levels of both transcripts were increased in monocytes engaged by immobilized anti-α_D_ mAb when compared to transcript levels in leukocytes incubated on immobilized IgG (2.4–9.2 fold), and were also increased (2.8-9.9 fold) when transcript levels in monocytes incubated on the immobilized anti-α_D_ mAb were compared to those in freshly-isolated monocytes from which mRNA was immediately extracted without incubation. Analysis by RT PCR confirmed that the mRNA for *ERα* was induced after a 1–4 hr incubation of monocytes on immobilized anti-α_D_ mAb 169B (Figure S4A). In three additional experiments, adhesion of monocytes on immobilized anti-α_D_ mAb 169B or mAb 217I induced expression of *IL-8* mRNA at 1-8 hr of incubation when examined by RT PCR, whereas the transcript was absent or expressed at lower levels in the cells at baseline and when incubated on immobilized IgG or HSA in parallel (Figure S4B-D). Together, these results confirmed the preliminary microarray findings.

**Figure 5:**
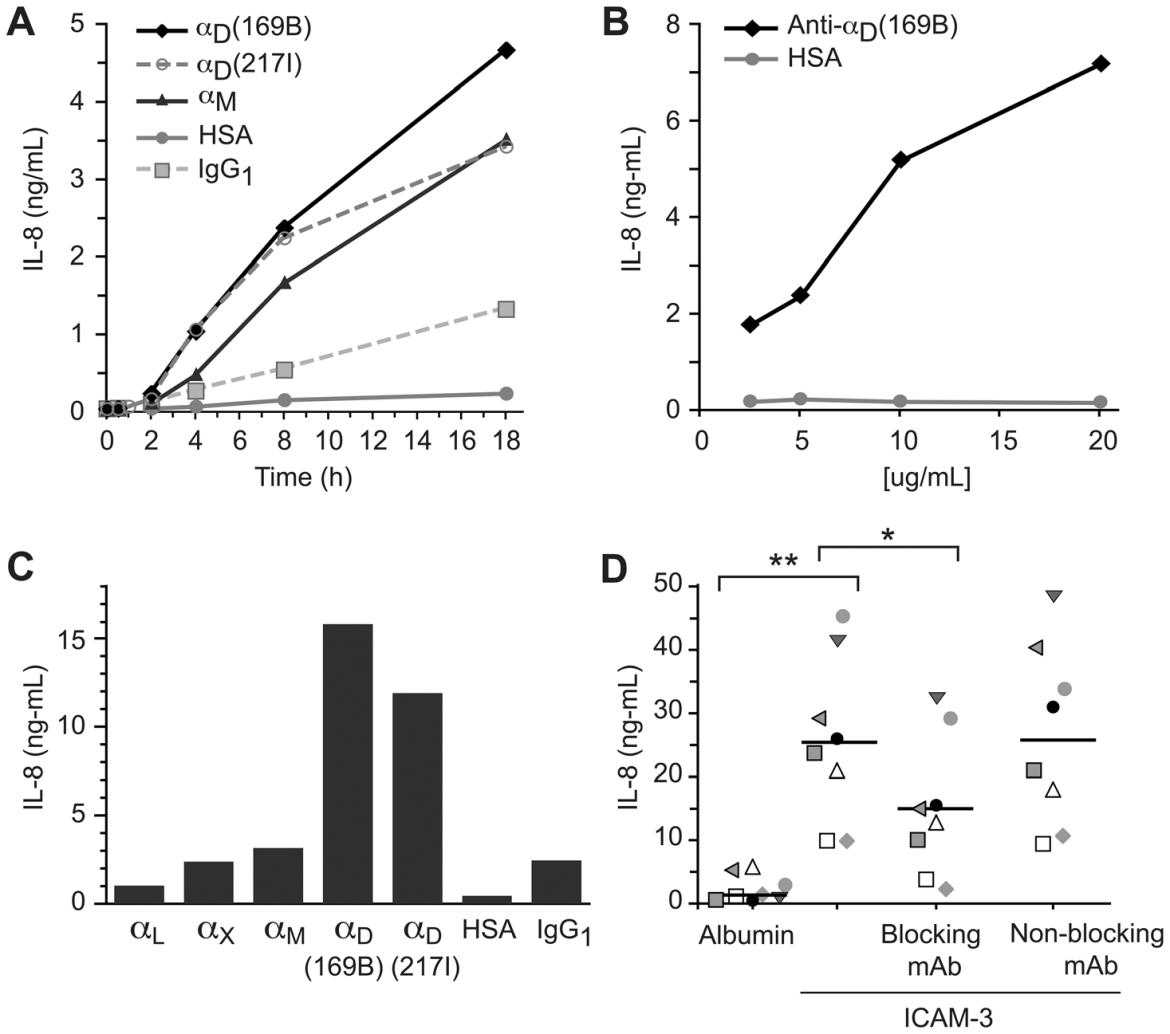
Engagement of integrin α_D_β_2_ induces expression and release of IL-8 by human monocytes. Unfractionated human monocytes were incubated in wells coated with immobilized mAb against α_D_, or in wells coated with HSA or IgG1 as control conditions, for various times. Supernatants were collected and analyzed for IL-8 by ELISA. In some experiments engagement of α_D_β_2_ was compared to engagement of other leukocyte integrins using immobilized mAb against individual leukocyte integrin α subunits. A. Engagement of α_D_β_2_ by immobilized anti-α_D_ mAb induced time-dependent release of IL-8, whereas IL-8 secretion by monocytes incubated on HSA- or IgG1-coated control surfaces was much lower. In parallel, incubation of monocytes with LPS (100 µg/ml) in suspension induced release of IL-8 at 8 (1.1 ng/ml) and 18 hr (4.4 ng/ml) (not shown). Engagement of αMβ_2_ also induced time-dependent release of IL-8. B. Release of IL-8 triggered by engagement of α_D_β_2_ on monocytes was dependent on the concentration of anti-α_D_ mAb used to coat the wells. This figure indicates the results from an 8 hr incubation of monocytes on the triggering and control surfaces. C. Engagement of integrin α_D_β_2_ was a potent stimulus for IL-8 secretion at 8 hr when immobilized mAb against α_D_ were compared to immobilized mAb against other leukocyte integrin α subunits. Two activating anti-α_D_ monoclonal antibodies, 169B and 217I, were examined in this experiment. The data in Panels A-C are individual determinations in single experiments. Data from additional experiments done at the 8 hr time point using monocytes from multiple different donors are shown in Tables S3 and S4. D. Wells were coated with human albumin or recombinant ICAM-3, monocytes were incubated in these wells for 18 hr. alone, in the presence of a blocking anti-α_D_ mAb (mAb 240I), or in the presence of a non-blocking anti-α_D_ monoclonal antibody (mAb 169A). IL-8 was then measured in the supernatants. The figure indicates the results of incubations with monocytes from 8 different subjects studied in 5 separate experiments on different days. Results from each subject are identified by a different symbol. The horizontal bars in the columns of data points indicate the means of determinations for each condition. The data were analyzed with Tukey's multiple comparison test and the Neuman-Kuels multiple comparison test, with similar results in each case. The significance values from the Neuman-Kuels analysis are shown (** = p<0.001; * = p<0.01). There was no difference in release of the IL-8 when monocytes were incubated with ICAM-3 alone versus incubation with ICAM-3 in the presence of the non-blocking mAb (p>0.05).

To provide additional validation for the physiologic relevance of these observations we further examined *IL-8* expression. Consistent with the mRNA analysis, in multiple experiments incubation of monocytes with immobilized anti-α_D_ activating mAb induced secretion of IL-8 protein (Figure 5A–C and Table S3). The magnitude of IL-8 release varied substantially from donor to donor, although release of IL-8 by monocytes from each subject was triggered by immobilized anti-α_D_ mAb. This feature is demonstrated in Table S3, which displays the raw data from 8 hr incubations in eight experiments. Secretion of IL-8 by monocytes engaged by the immobilized anti- α_D_ mAbs was consistently greater than that by cells incubated on immobilized HSA or IgG at 4–18 hr. (Figure 5A and Table S3). The magnitude of IL-8 release triggered by engagement of α_D_β_2_ was similar to that induced by LPS in parallel in a time course experiment (Figure 5A, legend). Release of IL-8 was dependent on the concentration of anti-α_D_ mAb used to coat the wells with immobilized antibody (Figure 5B). Immobilized anti-α_M_ mAb also induced expression of IL-8 protein with a magnitude that was variable (Figure 5A and Table S3). In three experiments, immobilized mAb against α_D_β_2_, α_M_β_2_, α_L_β_2_, and α_X_β_2_ were compared as triggers for outside-in signaling of IL-8 secretion. The magnitude of release of IL-8 by monocytes with specific leukocyte integrins engaged by the immobilized mAb was α_D_β_2_>α_M_β_2_≥α_X_β_2_≥α_L_β_2_ in each case (Figure 5C and Table S4).

Studies with primary leukocytes and transfected cell lines indicate that α_D_β_2_ recognizes a variety of ligands, including ICAM-3, vascular cell adhesion molecule 1 (VCAM-1), and several other adhesion and matrix factors [12],[14],[24],[30],[31]. ICAM-3 may be a preferred ligand under some conditions [12]. To further determine if engagement of α_D_β_2_ delivers outside-in signals, we coated plates with recombinant ICAM-3 or albumin, allowed monocytes to settle onto these surfaces, and measured release of IL-8 after an 18 hr. incubation period. In five separate experiments in which monocytes from eight healthy subjects were examined, there was enhanced release of IL-8 by monocytes adherent to ICAM-3 compared to that of monocytes in wells coated with albumin alone in each case (Figure 5D) (p<0.001), although the magnitude of secretion of IL-8 varied substantially from subject to subject. In parallel, a blocking anti-α_D_ mAb inhibited release of IL-8 by monocytes from each donor (p<0.01), whereas a non-blocking anti-α_D_ mAb did not inhibit IL-8 release or had only a trivial effect (p>0.05). The pattern of inhibition by the blocking anti-α_D_ mAb and lack of inhibition by the non-blocking mAb was consistent in incubations with monocytes from each of the eight subjects (Figure 5D). The blocking anti-α_D_ mAb did not completely inhibit IL-8 release, likely because ICAM-3 is also recognized by α_L_β_2_ [32], which is basally expressed by monocytes (Figure 1B). Nevertheless, these experiments demonstrate the engagement of α_D_β_2_ by a “natural ligand” induces outside-in signaling to inflammatory gene expression pathways, and complement those in which outside-in signaling by α_D_β_2_ was induced by activating anti-α_D_ antibodies (Figures 5A-C and Tables S3 and Table S4).

**Figure 6:**
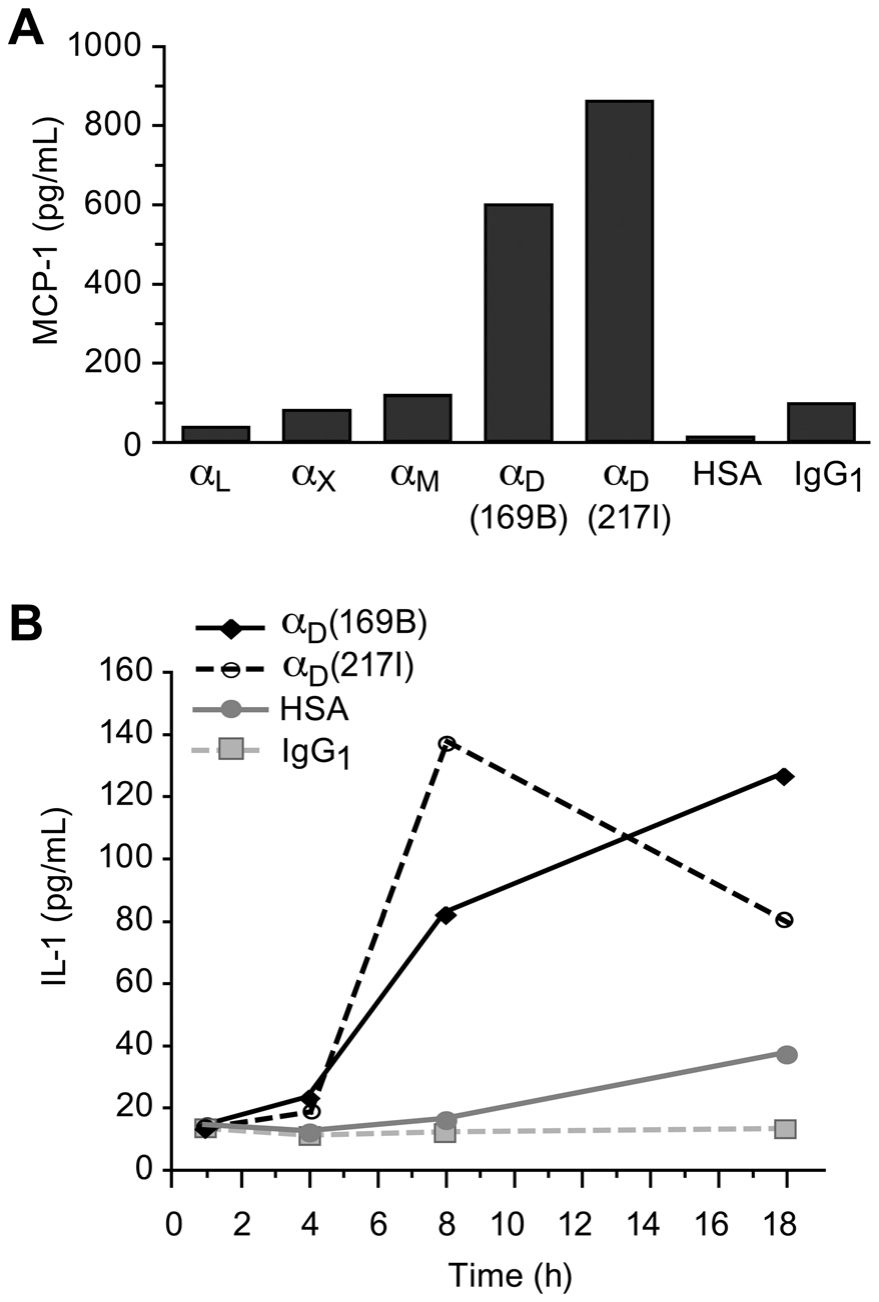
Engagement of integrin α_D_β_2_ triggers expression of inflammatory chemokines and cytokines by human monocytes. Integrin α_D_β_2_ was engaged by immobilized activating mAb and supernatants for mediator analysis were collected as outlined in Figure 5. In (A) the concentration of anti-α_D_ mAb, mAb against other leukocyte integrin α subunits, or control proteins used to coat the wells was 10 µg/ml, and in (B) the concentrations were 20 µg/ml. A. Engagement of integrin α_D_β_2_ (8 hr) triggered release of MCP-1 that was much greater than that induced by immobilized mAb against other leukocyte integrin α subunits or release from monocytes incubated on control surfaces. Two activating anti-α_D_ mAb, 169B and 217I, were studied. This result is representative of the pattern seen in eight separate experiments using monocytes from different donors, as shown in detail in Table S5. B. Engagement of integrin α_D_β_2_ induced time-dependent release of IL-1β by monocytes. Two activating anti-α_D_ mAb were examined, as in (A). In five additional experiments using monocytes from different donors, IL-1β protein was induced in monocyte lysates and released into the supernatants when integrin α_D_β_2_ was engaged for 8 hr., and was greater than that in samples from monocytes incubated on control surfaces (Table S6).

To determine if engagement of α_D_β_2_ triggers expression and release of other inflammatory mediators, we examined secretion of interleukin-1β (IL-1β) and monocyte chemotactic protein-1 (MCP-1). Expression of both factors is induced by outside-in signaling in adhesive interactions of human monocytes [36],[37],[54]. In multiple experiments, engagement of α_D_β_2_ by immobilized mAb triggered secretion of MCP-1 (Figure 6A and Table S5). As with secretion of IL-8 (Figure 5C), engagement by immobilized mAb against α_D_β_2_ was a more potent stimulus for MCP-1 release than was engagement of other leukocyte integrins (Figure 6A). Engagement of α_D_β_2_ also triggered cellular accumulation and release of IL-1β protein in a time-dependent fashion (Figure 6B and Table S6). The fraction of IL-1β that was released into solution varied in individual experiments (Table S6). Processing and release of IL-1β may be altered at multiple checkpoints when its expression is triggered by engagement of α_D_β_2_ since our microarray analysis suggested that mRNA for caspase 1, a key enzyme that influences processing and secretion of this cytokine [55], is also altered under these conditions (Table S2). This was confirmed in an experiment examining *caspase 1* mRNA by RT PCR.

## Discussion

There is confusion and potential controversy in the inflammation field regarding the expression of α_D_β_2_ on human leukocytes. Recent reviews and primary articles state that α_D_β_2_ is only expressed on tissue macrophages [56],[57], that it is principally expressed by eosinophils in humans [3], and that it is expressed “poorly” on human peripheral blood leukocytes but strongly on macrophage foam cells within atherosclerotic plaques [24],[25]. In contrast, however, the original characterization of human α_D_β_2_ reported that it is expressed on most unfractionated granulocytes and monocytes (94% and 98% respectively) isolated from peripheral blood samples and also on peripheral blood lymphocytes, although its expression levels varied on different leukocyte subsets and in relationship to other β_2_ integrins [12]. Our analysis in this study confirms that α_D_β_2_ is basally expressed on the majority of monocytes and neutrophils in whole venous blood and in isolated cell preparations, and on some peripheral blood lymphocytes (Figure 1A). Furthermore, we found that freshly-isolated human monocytes respond to engagement of α_D_β_2_ with functional alterations (Figures 5 and 6 and Tables S1, S2, S3, S4, S5 and S6), consistent with the presence of α_D_β_2_ on their surfaces (Figures 1A, B). In previous observations by other investigators α_D_β_2_ was reported to be expressed on human peripheral blood eosinophils [30], and to be upregulated on human peripheral blood granulocytes [12] and eosinophils [30] in response to stimulation. In a recent report α_D_ was detected on human neutrophils, NK cells, and 6-sulfo- LacNAc^+^ dendritic cells isolated from peripheral blood and cocultured in the presence of cytokines and LPS, although basal expression was apparently not examined [21]. While expression of the protein was not determined, messenger RNA for *α_D_* was detected in human peripheral blood monocytes in an earlier report [23]. In summary, all previous studies in which α_D_β_2_ or the *α_D_* transcript was specifically examined have indicated that α_D_β_2_ is expressed by human peripheral blood leukocytes or leukocyte subsets. Our observations here support this conclusion, confirm that α_D_β_2_ is displayed on the surfaces of human peripheral blood monocytes and neutrophils, and demonstrate that it alters monocyte functions by outside-in signaling when engaged.

The factors that influence expression of α_D_β_2_ by human leukocytes are not completely defined [23],[58],[59]. Analysis of a human genomic clone of *α_D_* (*ITGAD*) in cell lines indicated that a region of the promoter recognized by transcription factors Sp1 and Sp3 is sufficient to confer leukocyte-specific expression, and that kruppel-like factor 4 regulates expression of the *α_D_* gene in myeloid cells [58],[59]. Surface display may be regulated by extracellular domains of α_D_ [60].

Basal expression of α_D_β_2_ on a large fraction of circulating human leukocytes is different from its pattern of expression in mice [13],[14] and dogs [43], where it is restricted to a small percentage of leukocytes in blood under resting conditions. In some cases, it may be difficult to detect α_D_β_2_ on circulating leukocytes from uninfected or uninflamed mice at all [13], in contrast to its prominent display on circulating human myeloid cells (Figures 1 and 2). Furthermore, there are differences in the pattern of basal expression of α_D_β_2_ on resident tissue leukocytes of rodents and humans. It was not detected in the lungs of mice [14] and was present in only slight constitutive levels in lungs of rats [42] in the absence of inflammatory injury, whereas it is basally expressed and easily detectable on leukocytes in the uninjured human lung (Figure 3A). In these regards, differential expression of α_D_β_2_ is another example of variations in the immune biology of humans, mice, and other species [61]–[63]. The regulatory features that account for these inter-species differences are yet to be defined. Even though these differences are likely to have biologic importance, there are also similarities in expression of α_D_β_2_ on leukocytes of humans and experimental animals that argue for important conserved functions. It is highly expressed by macrophages in the splenic red pulp in humans, dogs, rats, and mice [12],[14],[17],[43], suggesting important recognition and clearance activities in defense against blood-borne pathogens and in surveillance of antigens and microparticulates [14]. Correlative studies of human leukocyte biology together with animal models may reveal unique functions of α_D_β_2_ in comparison to other leukocyte integrins [12], and explanations for similarities and divergence in its patterns of expression in rodents, dogs, and man.

In this study we focused on human monocytes and monocyte-derived immune effector cells for further characterization of α_D_β_2_ expression and activity. Together with the three other members of the leukocyte integrin family, α_D_β_2_ is expressed on circulating monocytes in the absence of stimulation or agonists for differentiation (Figures 1A, B, 2B, C). Although values for intensity of staining of each integrin were somewhat different, a similar pattern was also reported by van der Vieren et al. [12]. Studies with HL60 and THP-1 myeloid cell lines [12], which are leukemic in origin, indicate that α_D_β_2_ may also be expressed by neoplastic monocytes in humans. Both CD16^+^ and CD16^−^ monocyte subsets isolated from peripheral blood of healthy volunteers express α_D_β_2_ under basal conditions, consistent with its high level of constitutive display on unfractionated monocytes (Figures 1 and 2). It is proposed that CD14^+^ CD16^+^ monocytes preferentially home to the marginal pool of leukocytes in humans in part because of high basal expression of α_D_β_2_ [64]. Constitutive adhesive activity of leukocyte integrins as a mechanism of leukocyte margination under basal conditions is controversial [65]. Nevertheless, α_D_β_2_ is likely to contribute to targeting and trafficking of circulating human monocytes in inflammation, as it does for myeloid cells in rodents[18]–[20],[25].

Monocytes are parent cells for macrophages and MDDC [29],[40],[41], and we found that α_D_β_2_ is expressed by human monocyte-derived macrophages and dendritic cells subjected to a variety of culture conditions and inflammatory agonists (Figure 4, Table 1). This result is consistent with its expression on leukocytes in uninflamed and inflamed human lung (Figure 3). Integrin α_D_β_2_ is also expressed by lesional macrophages in clinical atherosclerosis [12] and by macrophages in uninflamed and inflamed human synovium [66]. Studies of *α_D_* mRNA expression by human leukocytes and leukocyte cell lines indicated that it is temporally expressed during macrophage differentiation, differentially regulated compared to other leukocyte integrin α subunits, and altered by lipoproteins and phorbol esters when macrophages are differentiated in vitro [23]. Integrin α_D_β_2_ is expressed by specific subsets of macrophages in mice with systemic infections, and *α_D_* mRNA and protein expression were induced during differentiation of a murine macrophage cell line in response to cytokine stimulation [14]. In studies in rats, there was only slight expression of α_D_ in lungs by western and immunocytochemical analysis under basal conditions – a pattern different from that in human lungs (Figure 3), as noted previously – but the mRNA and protein were rapidly and transiently induced in response to immune complex-stimulated alveolar inflammation [42]. The precise mechanisms that control dynamic regulation of α_D_β_2_ expression by macrophages and dendritic cells in inflamed human tissues and animal models remain to be defined, and may vary depending on the inflammatory conditions.

Integrin α_D_β_2_ mediates adhesion of primary human eosinophils [30], primary murine splenic macrophages [14], and a variety of cell lines and transfected cells [12],[24],[31]. Beyond this, little is known of the specific cellular functions of integrin α_D_β_2_. We found that engagement of α_D_β_2_ on human monocytes by some – but not all – immobilized mAb that recognize α_D_ induced dramatic cellular spreading (Table S1). In this approach, mAb specific for α_D_β_2_ were used as probes to determine if its engagement transmits outside-in signals, potentially mimicking engagement by ligands. Binding and crosslinking strategies utilizing antibodies against other surface adhesion molecules have previously been employed in a similar fashion [32],[50]–[53],[67]–[70]. Cellular spreading, as was induced by anti- α_D_ mAb in our experiments, is linked to gene expression pathways in a variety of cells including monocytes [32],[71]–[73]. Consistent with this, we found that engagement of α_D_β_2_ also alters the pattern of expressed genes by monocytes and induces release of inflammatory chemokines and cytokines.

Using a microarray approach as an initial screen, we documented that engagement of α_D_β_2_ by activating antibodies induces alterations in mRNA expression profiles of monocytes. Focusing first on transcripts that appeared to increase in monocytes in response to engagement of α_D_β_2_ identified by this preliminary approach, we confirmed increases in *ERα* and *IL-8*. *ERα* was chosen for validation not only because of the magnitude in alteration of its mRNA levels induced by α_D_β_2_ engagement (see “Results” and Table S2) but also because estrogen receptors have complex activities in inflammation and wound healing [74]–[76]. Similarly, IL-8 (CXCL8) has central activities in physiologic and pathologic inflammation [77] and the *IL-8* transcript and protein are frequently expressed in these conditions. These results provided a rationale for further experiments to explore regulation of synthesis of inflammatory proteins by α_D_β_2._ Focusing on IL-8, in multiple experiments we found that engagement of α_D_β_2_ induced release of this peptide chemokine (Figure 5 and Table S3, S4). We also found that IL-1β and MCP-1 are released under these conditions (Figure 6 and Table S5, S6). Together, these findings indicate that engagement of α_D_β_2_ delivers signals to pathways that control levels of transcripts for inflammatory genes and synthesis and release of inflammatory proteins by human monocytes. While in some cases engagement or co-engagement of Fc receptors is involved when activating antibodies are used as surrogate ligands to trigger adhesion molecule signaling [32],[50], control incubations with non-immune IgG (“Results”, Figures 5 and 6, Tables S3, S4, S5 and S6) support the conclusion that α_D_β_2_ has specific outside-in signal transduction capacity. Furthermore, we demonstrated that a purified ligand for α_D_β_2_, ICAM-3, induces outside-in signaling and chemokine release by monocytes that was inhibited by a blocking anti- α_D_ mAb (Figure 5D). Indirect experiments by other investigators suggest that α_D_β_2_ also potentiates the release of interferon gamma by human natural killer cells [21],[22]. Consistent with these observations, targeted deletion of α_D_ in mice – resulting in absence of α_D_β_2_ – yielded changes in cytokine and chemokine profiles in experimental models of malaria and *Salmonella* infection ([14]; manuscript submitted). The latter experiments suggest that outside-in signaling resulting from engagement of α_D_β_2_ regulates inflammatory chemokine and cytokine synthesis in vivo, as well as in vitro.

Previous reports indicate that engagement of β_2_ integrins induces altered inflammatory gene expression by human monocytes or neutrophils[51]–[53],[78]. Integrins may in some cases provide a “first signal” that induces mRNAs for a variety of inflammatory mediators when monocytes adhere to matrix proteins, while a “second signal” is required to trigger synthesis of the corresponding proteins [78]. Integrin α_M_β_2_ has been examined most in studies of outside-in signaling to inflammatory pathways, and we used engagement of α_M_β_2_ as a positive control in our analysis of α_D_β_2_ signaling. Engagement of α_D_β_2_ triggered release of IL-8, MCP-1, and IL-1β in levels that were equivalent to, or in many cases greater than, those induced by engagement of α_M_β_2_ (Figures 5, 6 and Tables S3, S4, S5 and S6). Integrins α_D_β_2_ and α_M_β_2_ share sequence homology and an overlapping pattern of ligands, but there is significant divergence in the sequence of their cytoplasmic tails (16% identity) [12],[24]. Differential interface of the cytoplasmic domains of α_D_β_2_ and α_M_β_2_ with key intracellular signal transduction pathways [48],[49] may dictate variations in expression and release of specific inflammatory gene products when the two integrins are ligated individually or together on the same leukocyte in vivo. This issue remains to be explored.

It is clear from this analysis that integrin α_D_β_2_ is significantly represented in the repertoire of surface adhesion molecules on circulating human myeloid leukocytes, in addition to expression on key human immune effector cells – macrophages and dendritic cells – that are localized in tissues. Like other members of the leukocyte integrin family, and similar to surface adhesion molecules such as P selectin glycoprotein 1 and ICAM-3 [26],[32],[37],[54], α_D_β_2_ mediates both adhesion and signaling. Thus, α_D_β_2_ may be important in complex cell-cell interactions involving circulating myeloid leukocytes, platelets, and endothelial cells [79],[80]. Furthermore, α_D_β_2_ is likely to have important, and perhaps unique [12], activities in human host defense and in inflammatory syndromes in which it has been detected on leukocytes in pathologic samples, including acute and chronic lung injury and inflammation (Figure 3; Miyazaki Y, unpublished observations), atherosclerosis [12], arthritis [66], and obesity [81]. Differences and similarities in α_D_β_2_ expression in humans and experimental animals may ultimately be exploited to further define these functions.

## Supporting Information

Figure S1 **Identification of α_D_β_2_-expressing leukocyte subpopulations in whole blood by flow cytometry.** Monocytes, neutrophils, and T-lymphocytes were identified as detailed in Materials and Methods. The panels illustrate gated cell populations in the same venous blood sample from a healthy volunteer donor. **A**) Gated monocytes (3.9% of total white blood cells) were labeled with fluorescently-conjugated anti-CD14 (FL1-height) and had expected side scatter characteristics. **B**) The gated neutrophil population (64.1% of total white blood cells in this sample) were labeled with fluorescent anti-CD15 and had an expected side scatter profile. **C**) The gated T-lymphocyte population (19.4% of total white blood cells in this sample) were identified by labeled anti-CD3 and had an appropriate side scatter profile. Each of the gated populations was evaluated for co-expression of α_D_ using alexa fluor 647-conjugated anti-α_D_ mAb 169A. Analyses of leukocytes in venous blood samples from four individual subjects using this approach are summarized in main text Figure 1A.
(JPG)

Figure S2 **CD16-negative and CD16-positive human monocyte subpopulations express α_D_β_2_.** CD16^−^ and CD16^+^ monocytes were isolated, stained with alexa fluor 647-conjugated anti-α_D_ mAb 169A, and examined by fluorescent microscopy as outlined in Materials and Methods. In parallel, monocytes of each subtype were incubated with isotype-specific non-immune IgG as a control for the anti-α_D_ mAb. **A, C**) IgG controls; **B, D**) staining with anti-α_D_ mAb 169A.
(JPG)

Figure S3 **Immunofluorescent staining of α_D_β_2_ on human neutrophils (PMNs).** Isolated neutrophils were suspended in Hanks Balanced Salt Solution with albumin (HBSS/A) and incubated with anti-α_D_ mAbs 169A, 169B, 217I, or 240I or with non-immune mouse IgG_1_ (all immunoglobulins at 10 µg/mL final concentration) at 37° for 45 min. The cells were then centrifuged (1,000xg at 4°C for 5 min), resuspended in HBSS/A, and incubated with isotonic 8% paraformaldehyde/4% sucrose in phosphate-buffered saline for 20 min at 4°C. The PMNs were then washed three times with HBSS/A and incubated with FITC-conjugated goat anti-mouse IgG (Molecular Probes) (2 µg/mL) for 30 min on ice, followed by washing with HBSS/A and examination by confocal microscopy. Staining with the anti-α_D_ mAbs suggested plasma membrane clustering of integrin α_D_β_2_. Incubation with non-immune IgG_1_ yielded no fluorescent staining and was not photographed.
(JPG)

Figure S4 **Induction of transcripts for Estrogen Receptor α and Interleukin 8 (IL-8) in human monocytes by activating anti-α_D_ mAb 169A and 217I.** Wells were coated with the activating anti-α_D_ or control immunoglobulins and proteins and isolated human monocytes were incubated on these surfaces as described in the legends to Figure 5 and Table S3. Cellular lysates were then probed for transcripts for estrogen receptor α, IL-8, and GADPH by polymerase chain reaction. **A-C**) In these individual experiments monocytes were incubated on immobilized mAb 169B for the times shown, followed by PCR analysis. **D**) In this experiment human monocytes were incubated on immobilized activating anti-α_D_ mAb 169B or 217I, anti-α_M_, human serum albumin (HSA), or non-immune IgG_1_ for 8 hr followed by PCR analysis. Co = control: no sample added.
(JPG)

Table S1 **Spreading of human monocytes on immobilized anti-α_D_ mAbs or control immunoglobulins or proteins.** Wells were coated with anti-α_D_ mAb, anti-α_M_, human serum albumin (HSA), or non-immune murine IgG1 (10 µg/mL for each immunoglobulin or protein) and isolated human monocytes were added and incubated for the specified times. The fraction of spread cells was determined by microscopy and counting.
(DOCX)

Table S2 **Microarray analysis of transcripts altered in human monocytes incubated on immobilized anti-α_D_ mAb 169B compared to freshly-isolated monocytes or monocytes incubated on immobilized control IgG1.** This summary lists transcripts that were coordinately increased or decreased when expression levels in monocytes incubated on immobilized anti-α_D_ mAb 169B were compared to expression in monocytes incubated in wells coated with non-immune IgG and to expression in freshly isolated monocytes. In this experiment, freshly-isolated monocytes suspended in medium199 with polymyxin B (1 µg/mL) were incubated with immobilized mAb 169B or IgG1 for 2 hr. The monocytes were then scraped from the wells, collected in Trizol, and stored at −70°C. In parallel, equal numbers of the freshly-isolated monocytes were collected in Trizol without incubation and frozen at -70°C. Microarray analysis of expressed transcripts was done as described.
(DOCX)

Table S3 **Incubation of human monocytes on immobilized activating anti-α_D_ antibodies 169B and 217I induces release of interleukin 8 (IL-8).** Wells were coated with anti-α_D_ mAb 169B or 217I, anti-α_M_, human serum albumin (HSA), or non-immune IgG1 (10 µg/mL for all immunoglobulins and proteins) at 4° overnight and washed. Isolated human monocytes suspended in medium 199 containing polymyxin B (1 µg/mL) were added and incubated for 8 hr at 37° in 5% CO_2_, 95% air. The supernatants were removed from the wells, centrifuged (15,800 xg, 5 min), and stored at −70°. IL-8 and MCP-1 (see Table S5) in the supernatants were later measured by ELISA. The values are in pg/mL. Although there was substantial variation among individual donors in the eight experiments, in each case release of IL-8 from monocytes adherent to immobilized anti-α_D_ mAbs 169B and 217I was greater than that from monocytes incubated on the control proteins. In three of these experiments anti-α_X_ and anti-α_L_ were also examined in comparison to the anti-α_D_ mAb, anti-α_M_, and control proteins (see Table S4).
(DOCX)

Table S4 **Release of IL-8 by monocytes incubated on immobilized stimulating anti-α_D_ mAbs 169B and 217I, antibodies against α_M_, α_X_, or α_L_, or control protein surfaces.** Parallel incubations of monocytes for 8 hr in wells coated with human serum albumin (HSA), non-immune IgG1, anti-α_D_ mAbs 169B or 217I, or anti-α_M_, anti-α_X_, or anti-α_L_ were done as described in Table S3. At the end of the incubation supernatants were collected, processed, and assayed as outlined in Table S3. The values for IL-8 are in pg/mL.
(DOCX)

Table S5 **Incubation of human monocytes on immobilized activating anti-α_D_ antibodies (mAb 169B, 217I) induces release of MCP-1.** Wells were coated with anti-α_D_ mAb 169B or 217I, anti-α_M_, human serum albumin (HSA), or non-immune IgG1 and isolated human monocytes were incubated with these immobilized immunoglobulins and proteins as described in the legend for Table S3. Supernatants were collected at the end of an 8 hr incubation, frozen at −70°, and MCP-1 in the supernatants was later measured by ELISA. The values are in pg/mL. In each experiment, the concentration of MCP-1 was higher in supernatants from monocytes incubated on immobilized anti-α_D_ mAb 169B and 217I than in supernatants from monocytes incubated on control surfaces, although the values varied substantially among individual donors in the 8 experiments.
(DOCX)

Table S6 **Expression of IL-1β in human monocytes incubated on immobilized activating anti-α_D_ mAb or control proteins.** Isolated human monocytes were incubated on immobilized anti-α_D_ mAb 169B or 217I, anti-α_M_, human serum albumin (HSA), or non-immune IgG1 as described in Table S3. Supernatants were collected after an 8 hr. incubation and centrifuged as in Table S3. The adherent monocytes were then scraped from the wells, pooled with pellets from centrifugation of the supernatants, and lysed. The supernatants and monocyte lysates were stored separately at -70°C and later assayed for IL-1β by ELISA. The values shown are in pg/mL.
(DOCX)
