# Supplementary figures and images for: Integrin αDβ2 (CD11d/CD18) Is Expressed by Human Circulating and Tissue Myeloid Leukocytes and Mediates Inflammatory Signaling
Source: PLoS One. 2014 Nov 21;9(11):e112770. doi: 10.1371/journal.pone.0112770 (PMC4240710; doi:10.1371/journal.pone.0112770)

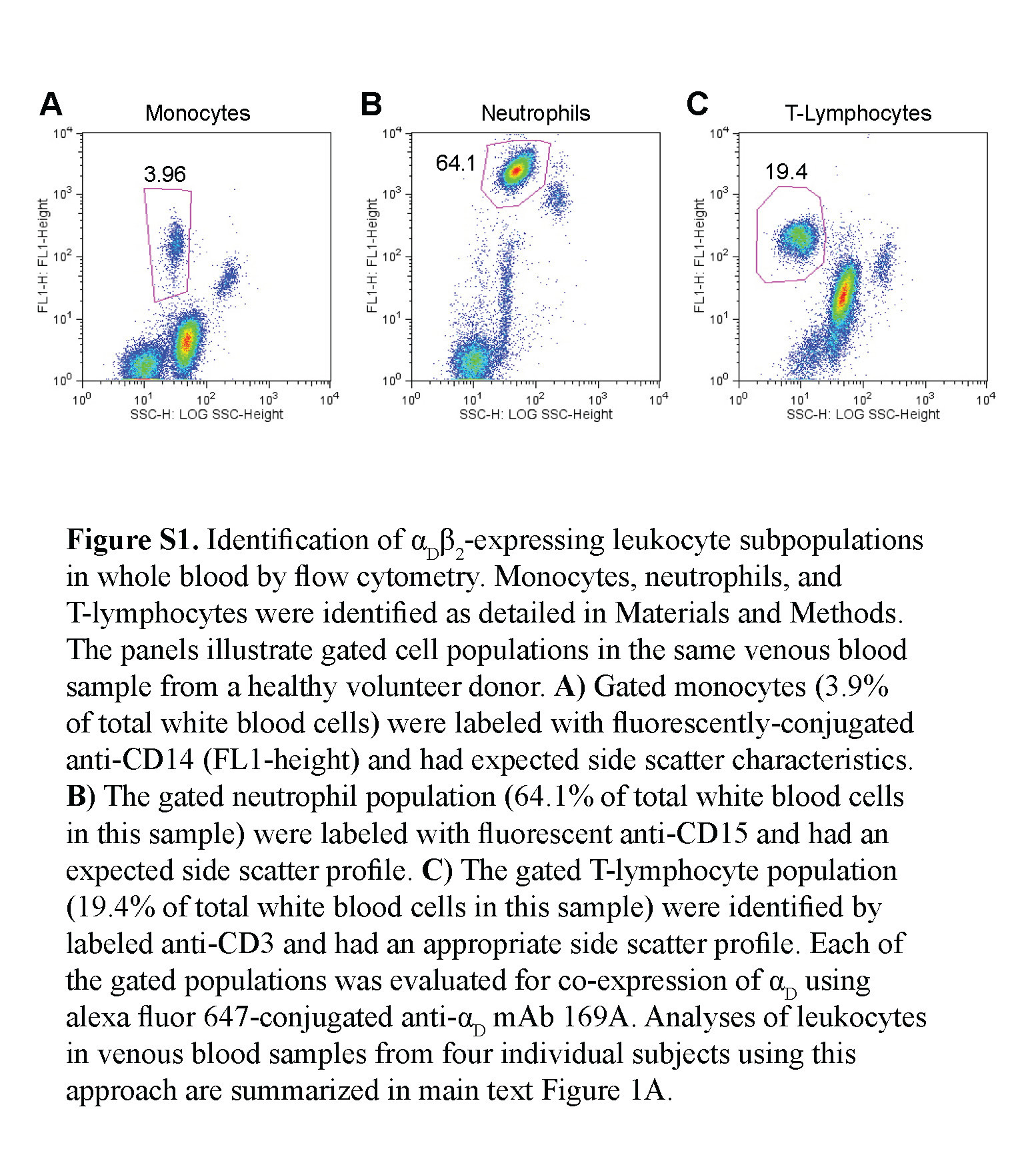

Supplement: Figure S1 — Identification of αDβ2-expressing leukocyte subpopulations in whole blood by flow cytometry. Monocytes, neutrophils, and T-lymphocytes were identified as detailed in Materials and Methods. The panels illustrate gated cell populations in the same venous blood sample from a healthy volunteer donor. A) Gated monocytes (3.9% of total white blood cells) were labeled with fluorescently-conjugated anti-CD14 (FL1-height) and had expected side scatter characteristics. B) The gated neutrophil population (64.1% of total white blood cells in this sample) were labeled with fluorescent anti-CD15 and had an expected side scatter profile. C) The gated T-lymphocyte population (19.4% of total white blood cells in this sample) were identified by labeled anti-CD3 and had an appropriate side scatter profile. Each of the gated populations was evaluated for co-expression of αD using alexa fluor 647-conjugated anti-αD mAb 169A. Analyses of leukocytes in venous blood samples from four individual subjects using this approach are summarized in main text Figure 1A. (JPG) [file pone.0112770.s001.jpg]

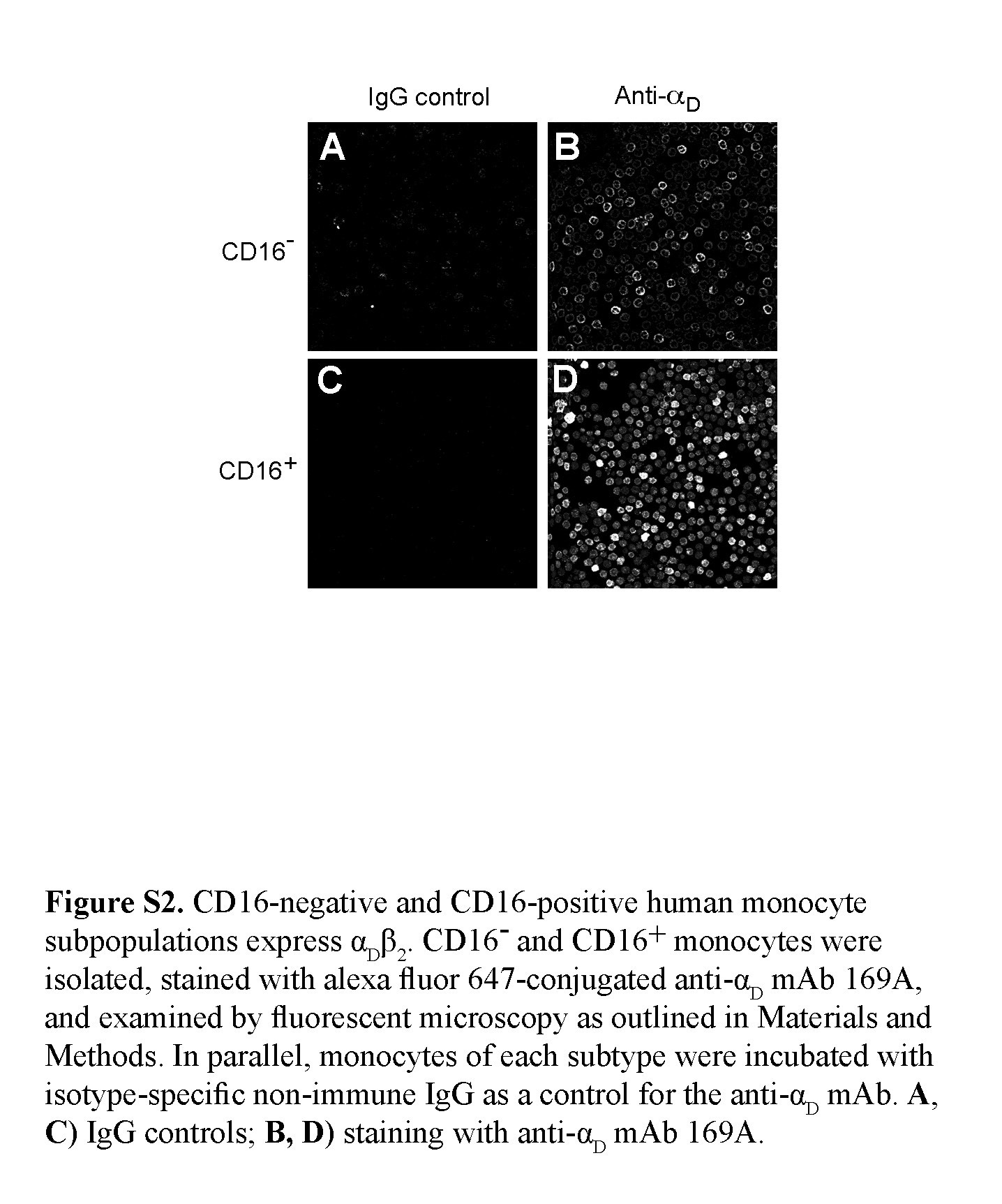

Supplement: Figure S2 — CD16-negative and CD16-positive human monocyte subpopulations express αDβ2. CD16− and CD16+ monocytes were isolated, stained with alexa fluor 647-conjugated anti-αD mAb 169A, and examined by fluorescent microscopy as outlined in Materials and Methods. In parallel, monocytes of each subtype were incubated with isotype-specific non-immune IgG as a control for the anti-αD mAb. A, C) IgG controls; B, D) staining with anti-αD mAb 169A. (JPG) [file pone.0112770.s002.jpg]

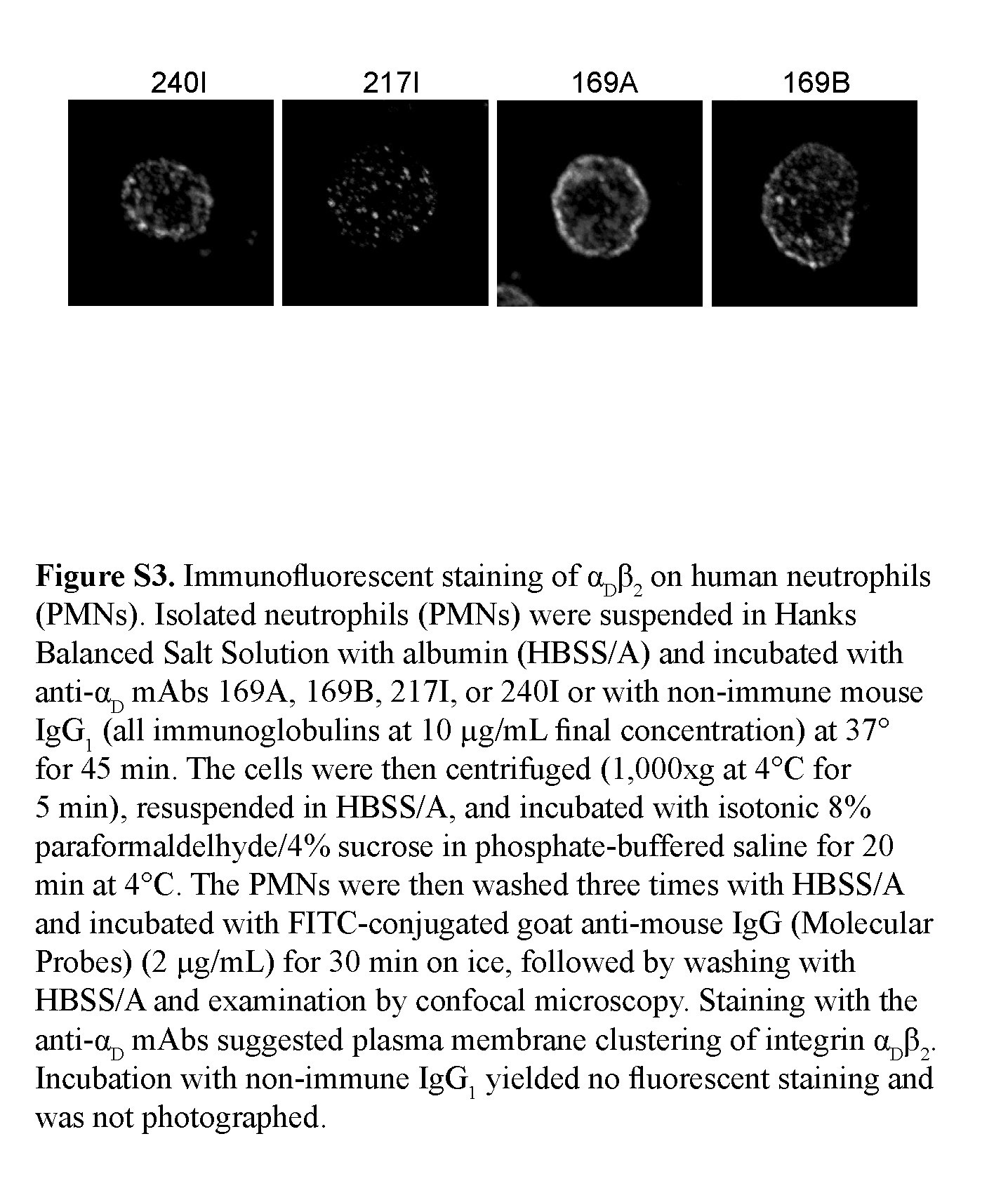

Supplement: Figure S3 — Immunofluorescent staining of αDβ2 on human neutrophils (PMNs). Isolated neutrophils were suspended in Hanks Balanced Salt Solution with albumin (HBSS/A) and incubated with anti-αD mAbs 169A, 169B, 217I, or 240I or with non-immune mouse IgG1 (all immunoglobulins at 10 µg/mL final concentration) at 37° for 45 min. The cells were then centrifuged (1,000xg at 4°C for 5 min), resuspended in HBSS/A, and incubated with isotonic 8% paraformaldehyde/4% sucrose in phosphate-buffered saline for 20 min at 4°C. The PMNs were then washed three times with HBSS/A and incubated with FITC-conjugated goat anti-mouse IgG (Molecular Probes) (2 µg/mL) for 30 min on ice, followed by washing with HBSS/A and examination by confocal microscopy. Staining with the anti-αD mAbs suggested plasma membrane clustering of integrin αDβ2. Incubation with non-immune IgG1 yielded no fluorescent staining and was not photographed. (JPG) [file pone.0112770.s003.jpg]

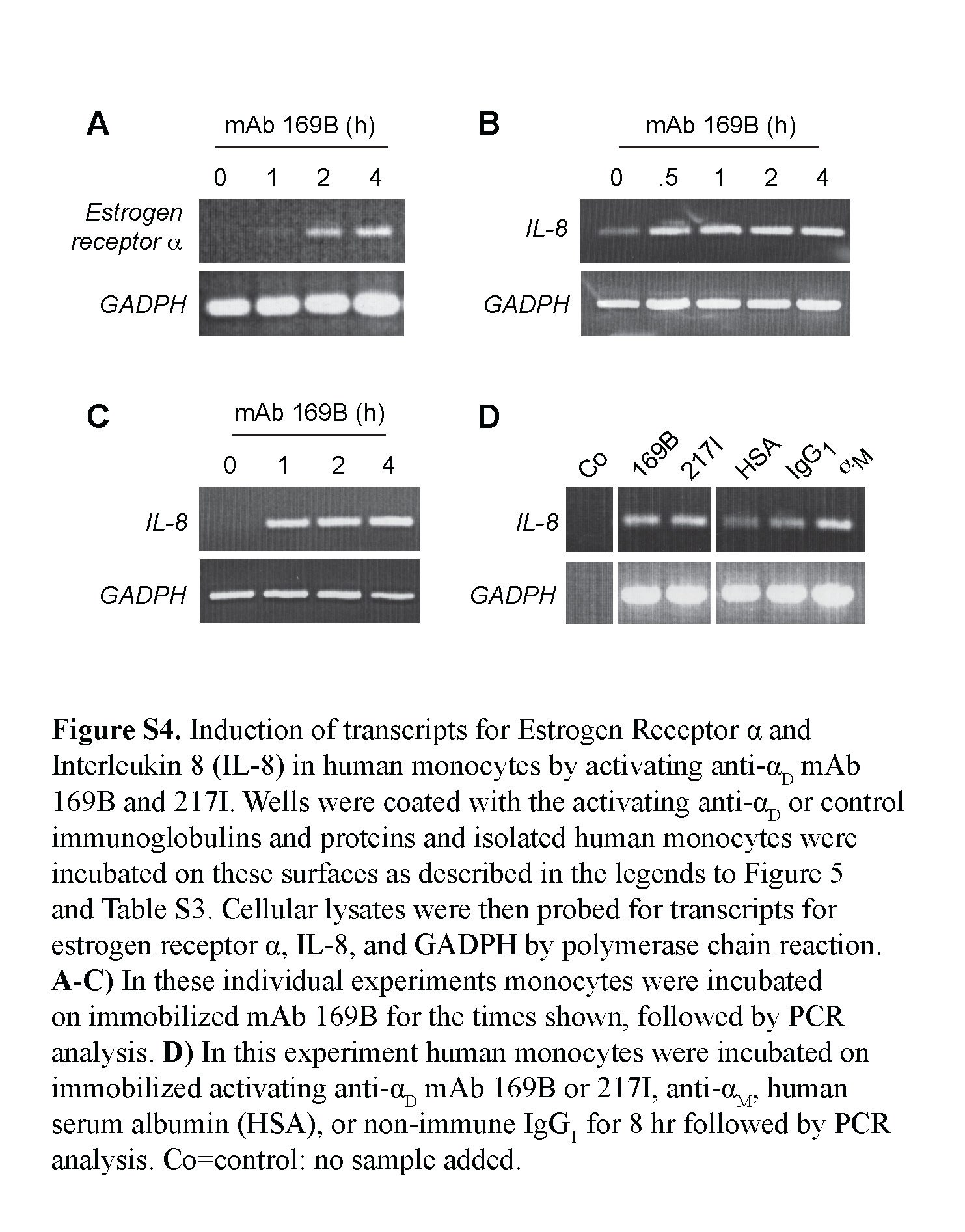

Supplement: Figure S4 — Induction of transcripts for Estrogen Receptor α and Interleukin 8 (IL-8) in human monocytes by activating anti-αD mAb 169A and 217I. Wells were coated with the activating anti-αD or control immunoglobulins and proteins and isolated human monocytes were incubated on these surfaces as described in the legends to Figure 5 and Table S3. Cellular lysates were then probed for transcripts for estrogen receptor α, IL-8, and GADPH by polymerase chain reaction. A-C) In these individual experiments monocytes were incubated on immobilized mAb 169B for the times shown, followed by PCR analysis. D) In this experiment human monocytes were incubated on immobilized activating anti-αD mAb 169B or 217I, anti-αM, human serum albumin (HSA), or non-immune IgG1 for 8 hr followed by PCR analysis. Co = control: no sample added. (JPG) [file pone.0112770.s004.jpg]
