# Supplementary material for: Integrin αDβ2 (CD11d/CD18) Is Expressed by Human Circulating and Tissue Myeloid Leukocytes and Mediates Inflammatory Signaling
Source: PLoS One. 2014 Nov 21;9(11):e112770. doi: 10.1371/journal.pone.0112770 (PMC4240710; doi:10.1371/journal.pone.0112770)
Supplement: Table S1 — Spreading of human monocytes on immobilized anti-αD mAbs or control immunoglobulins or proteins. Wells were coated with anti-αD mAb, anti-αM, human serum albumin (HSA), or non-immune murine IgG1 (10 µg/mL for each immunoglobulin or protein) and isolated human monocytes were added and incubated for the specified times. The fraction of spread cells was determined by microscopy and counting. (DOCX) [file pone.0112770.s005.docx]

**Supporting Information**

| **Table S1: Spreading of human monocytes on immobilized anti-α_D_  mAbs or control immunoglobulins or proteins** | | | | | | | | | | |
| --- | --- | --- | --- | --- | --- | --- | --- | --- | --- | --- |
|  | | | | | | | | | | |
| Experiment 1 |  | **α_D_(169B)** | **α_D_(217I)** | α_D_(169A) | α_D_(212D) | α_D_(217L) | α_D_(240I) | α_M_ | HSA | IgG1 |
|  | 30 min | **50%** | **15%** | 5% | 10% | 10% | 5% | 20% | 5% | 10% |
|  | 1 hr | **80%** | **40%** | 5% | 5% | 5% | 5% | 60% | 20% | 20% |
|  | 2 hr | **90%** | **40%** | 20% | 10% | 15% | 20% | 90% | 10% | 20% |
|  | 8 hr | **90%** | **60%** | 20% | 40% | 15% | 20% | 90% | 20% | 60% |
|  |  |  |  |  |  |  |  |  |  |  |
|  |  |  |  |  |  |  |  |  |  |  |
|  |  |  |  |  |  |  |  |  |  |  |
| Experiment 2 |  | **α_D_(169B)** | **α_D_(217I)** | α_D_(169A) | α_D_(212D) | α_D_(217L) | α_D_(240I) | α_M_ | HSA | IgG1 |
|  | 5 min | **5%** | **5%** | 5% | 5% | 5% | 5% | 10% | 0% | 5% |
|  | 15 min | **40%** | **40%** | 15% | 40% | 10% | 15% | 50% | 10% | 10% |
|  | 30 min | **40%** | **40%** | 10% | 30% | 10% | 40% | 50% | 10% | 30% |
|  | 1 hr | **70%** | **50%** | 20% | 20% | 20% | 20% | 60% | 5% | 30% |
|  | 2 hr | **80%** | **70%** | 10% | 20% | 30% | 20% | 60% | 5% | 10% |
|  | 8 hr | **80%** | **70%** | 10% | 20% | 20% | 70% | 70% | 5% | 40% |
|  |  |  |  |  |  |  |  |  |  |  |
|  |  |  |  |  |  |  |  |  |  |  |
| Mean Values for the Two Experiments |  | **α_D_(169B)** | **α_D_(217I)** | α_D_(169A) | α_D_(212D) | α_D_(217L) | α_D_(240I) | α_M_ | HSA | IgG1 |
|  | 30 min | **45%** | **27.5%** | 7.5% | 20% | 10% | 22.5% | 35% | 7.5% | 20% |
|  | 1 hr | **75%** | **45%** | 12.5% | 12.5% | 12.5% | 12.5% | 60% | 12.5% | 25% |
|  | 2 hr | **85%** | **55%** | 15% | 15% | 22.5% | 20% | 75% | 7.5% | 15% |
|  | 8 hr | **85%** | **65%** | 15% | 30% | 17.5% | 45% | 80% | 12.5% | 50% |

Table S1 Legend: Wells were coated with anti-α_D_ mAb, anti-α_M_, human serum albumin (HSA), or non-immune murine IgG1 (10 µg/mL for each immunoglobulin or protein) and isolated human monocytes were added and incubated for the specified times. The fraction of spread cells was determined by microscopy and counting (32).
