# Supplementary material for: Integrin αDβ2 (CD11d/CD18) Is Expressed by Human Circulating and Tissue Myeloid Leukocytes and Mediates Inflammatory Signaling
Source: PLoS One. 2014 Nov 21;9(11):e112770. doi: 10.1371/journal.pone.0112770 (PMC4240710; doi:10.1371/journal.pone.0112770)
Supplement: Table S2 — Microarray analysis of transcripts altered in human monocytes incubated on immobilized anti-αD mAb 169B compared to freshly-isolated monocytes or monocytes incubated on immobilized control IgG1. This summary lists transcripts that were coordinately increased or decreased when expression levels in monocytes incubated on immobilized anti-αD mAb 169B were compared to expression in monocytes incubated in wells coated with non-immune IgG and to expression in freshly isolated monocytes. In this experiment, freshly-isolated monocytes suspended in medium199 with polymyxin B (1 µg/mL) were incubated with immobilized mAb 169B or IgG1 for 2 hr. The monocytes were then scraped from the wells, collected in Trizol, and stored at −70°C. In parallel, equal numbers of the freshly-isolated monocytes were collected in Trizol without incubation and frozen at -70°C. Microarray analysis of expressed transcripts was done as described. (DOCX) [file pone.0112770.s006.docx]

| **Table S2: Microarray analysis of transcripts altered in human monocytes incubated on immobilized anti-α_D_ mAb 169B compared to freshly-isolated monocytes or monocytes incubated on immobilized control IgG1** | | | |
| --- | --- | --- | --- |
|  |  |  | |
| Transcript | Ratio of Transcript Expression in Monocytes Incubated on Immobilized mAb 169B Compared to Monocytes Incubated on IgG1 | | Ratio of Transcript Expression in Monocytes Incubated on Immobilized mAb 169B Compared to Freshly-Isolated Monocytes |
| **Increased** |  | |  |
| *Interleukin 8* | 2.4 | | 9.9 |
| *Estrogen receptor α* | 8.1 | | 2.8 |
| *Monocyte Chemotactic Protein 1* | 2.0 | | 2.7 |
| *Death Associated Protein 3* | 2.2 | | 2.5 |
| *Gelsolin* | 3.2 | | 2.7 |
| *Mitofilin* | 2.2 | | 2.3 |
| *Interferon-Induced Protein 35* | 3.1 | | 2.0 |
| *Macrophage Stimulating Receptor 1* | 2.0 | | 3.0 |
| *Single-stranded DNA-binding Protein* | 3.2 | | 2.9 |
| *Solute Carrier Family (Organic Ion Transporter) Member 3* | 2.3 | | 2.2 |
| *Survival Motor Neuron 1* | 3.0 | | 2.1 |
| *alpha 2A amylase* | 5.9 | | 9.5 |
| **Decreased** |  | |  |
| *Caspase 1 (Interleukin 1 β Convertase)* | 0.3 | | 0.3 |
| *High-Mobility Group Protein 2* | 0.5 | | 0.4 |
| *Interleukin 4 Receptor* | 0.3 | | 0.5 |
| *Leukocyte Immunoglobulin-Like Receptor* | 0.4 | | 0.4 |
| *Low Density Lipoprotein-Related Receptor 2* | 0.4 | | 0.4 |
| *Lysyl Oxidase* | 0.3 | | 0.4 |
| *alpha mannosidase, Class 2A, Member 2* | 0.5 | | 0.5 |
| *Mitogen-activated Protein Kinase 6* | 0.4 | | 0.5 |
| *Protein Tyrosine Phosphatase, receptor-type, Zeta Polypeptide 1* | 0.5 | | 0.5 |
| *Receptor Tyrosine Kinase-like Orphan Receptor 2* | 0.5 | | 0.5 |
| *Succinate Dehydrogenase Complex, Subunit A* | 0.5 | | 0.4 |
| *Soluble Superoxide Dismutase 1* | 0.5 | | 0.4 |

Table S2 Legend: This summary lists transcripts that were coordinately increased or decreased when expression levels in monocytes incubated on immobilized anti-α_D_ mAb 169B were compared to expression in monocytes incubated in wells coated with non-immune IgG and to expression in freshly isolated monocytes. In this experiment, freshly-isolated monocytes suspended in medium199 with polymyxin B (1 µg/mL) were incubated with immobilized mAb 169B or IgG1 for 2 hr. The monocytes were then scraped from the wells, collected in Trizol, and stored at -70°C. In parallel, equal numbers of the freshly-isolated monocytes were collected in Trizol without incubation and frozen at -70°C. Microarray analysis of expressed transcripts was done as described (35, 37).
