# Supplementary material for: Integrin αDβ2 (CD11d/CD18) Is Expressed by Human Circulating and Tissue Myeloid Leukocytes and Mediates Inflammatory Signaling
Source: PLoS One. 2014 Nov 21;9(11):e112770. doi: 10.1371/journal.pone.0112770 (PMC4240710; doi:10.1371/journal.pone.0112770)
Supplement: Table S3 — Incubation of human monocytes on immobilized activating anti-αD antibodies 169B and 217I induces release of interleukin 8 (IL-8). Wells were coated with anti-αD mAb 169B or 217I, anti-αM, human serum albumin (HSA), or non-immune IgG1 (10 µg/mL for all immunoglobulins and proteins) at 4° overnight and washed. Isolated human monocytes suspended in medium 199 containing polymyxin B (1 µg/mL) were added and incubated for 8 hr at 37° in 5% CO2, 95% air. The supernatants were removed from the wells, centrifuged (15,800 xg, 5 min), and stored at −70°. IL-8 and MCP-1 (see Table S5) in the supernatants were later measured by ELISA. The values are in pg/mL. Although there was substantial variation among individual donors in the eight experiments, in each case release of IL-8 from monocytes adherent to immobilized anti-αD mAbs 169B and 217I was greater than that from monocytes incubated on the control proteins. In three of these experiments anti-αX and anti-αL were also examined in comparison to the anti-αD mAb, anti-αM, and control proteins (see Table S4). (DOCX) [file pone.0112770.s007.docx]

| **Table S3: Incubation of human monocytes on immobilized activating anti-α_D_ antibodies 169B and 217I induces release of interleukin 8 (IL-8)** | | | | | |
| --- | --- | --- | --- | --- | --- |
| Experiment | HSA | IgG1 | **mAb 169B** | **mAb 217I** | anti-α_M_ |
| 1 | 166 | 2292 | **8236** | **5500** | 1177 |
| 2 | 483 | 1437 | **5225** | **3289** | 1425 |
| 3 | 97 | 782 | **7253** | **5033** | 631 |
| 4 | 117 | 1039 | **3091** | **3091** | 493 |
| 5 | 196 | 1050 | **1707** | **1614** | 755 |
| 6 | 60 | 298 | **2019** | **1205** | 437 |
| 7 | 205 | 2459 | **15,911** | **11,984** | 3170 |
| 8 | 181 | 792 | **5510** | **2848** | 700 |
| Mean | 188 | 1269 | **6119** | **4316** | 1099 |
| Range | 60-483 | 298-2459 | **1707-15,911** | **1205-11,984** | 437-3170 |
| Table S3 Legend: Wells were coated with anti-α_D_ mAb 169B or 217I, anti-α_M_, human serum albumin (HSA), or non-immune IgG1 (10 µg/mL for all immunoglobulins and proteins) at 4° overnight and washed. Isolated human monocytes suspended in medium 199 containing polymyxin B (1 µg/mL) were added and incubated for 8 hr at 37° in 5% CO_2_, 95% air. The supernatants were removed from the wells, centrifuged (15,800 xg, 5 min), and stored at -70°. IL-8 and MCP-1 (see Table S5) in the supernatants were later measured by ELISA. The values are in pg/mL. Although there was substantial variation among individual donors in the eight experiments, in each case release of IL-8 from monocytes adherent to immobilized anti-α_D_ mAbs 169B and 217I was greater than that from monocytes incubated on the control proteins. In three of these experiments anti-α_X_ and anti-α_L_ were also examined in comparison to the anti-α_D_ mAb, anti-α_M_, and control proteins (see Table S4). | | | | | |
