# Supplementary material for: Integrin αDβ2 (CD11d/CD18) Is Expressed by Human Circulating and Tissue Myeloid Leukocytes and Mediates Inflammatory Signaling
Source: PLoS One. 2014 Nov 21;9(11):e112770. doi: 10.1371/journal.pone.0112770 (PMC4240710; doi:10.1371/journal.pone.0112770)
Supplement: Table S4 — Release of IL-8 by monocytes incubated on immobilized stimulating anti-αD mAbs 169B and 217I, antibodies against αM, αX, or αL, or control protein surfaces. Parallel incubations of monocytes for 8 hr in wells coated with human serum albumin (HSA), non-immune IgG1, anti-αD mAbs 169B or 217I, or anti-αM, anti-αX, or anti-αL were done as described in Table S3. At the end of the incubation supernatants were collected, processed, and assayed as outlined in Table S3. The values for IL-8 are in pg/mL. (DOCX) [file pone.0112770.s008.docx]

| **Table S4: Release of IL-8 by monocytes incubated on immobilized stimulating anti-α_D_ mAbs 169B and 217I, antibodies against α_M_, α_X_, or α_L_, or control protein surfaces** | | | | | | | |
| --- | --- | --- | --- | --- | --- | --- | --- |
| Experiment | HSA | IgG1 | **mAb 169B** | **mAb 217I** | anti-α_M_ | anti-α_X_ | anti-α_L_ |
| 1 | 60 | 298 | **2019** | **1205** | 437 | 471 | 391 |
| 2 | 205 | 2459 | **15,911** | **11,984** | 3170 | 2397 | 1037 |
| 3 | 181 | 792 | **5510** | **2848** | 700 | 391 | 666 |
| Mean | 149 | 1183 | **7813** | **5346** | 1436 | 1086 | 698 |

Table S4 Legend: Parallel incubations of monocytes for 8 hr in wells coated with human serum albumin (HSA), non-immune IgG1, anti-α_D_ mAbs 169B or 217I, or anti-α_M_, anti-α_X_, or anti-α_L_ were done as described in Table S3. At the end of the incubation supernatants were collected, processed, and assayed as outlined in Table S3. The values for IL-8 are in pg/mL.
