# Supplementary material for: Integrin αDβ2 (CD11d/CD18) Is Expressed by Human Circulating and Tissue Myeloid Leukocytes and Mediates Inflammatory Signaling
Source: PLoS One. 2014 Nov 21;9(11):e112770. doi: 10.1371/journal.pone.0112770 (PMC4240710; doi:10.1371/journal.pone.0112770)
Supplement: Table S5 — Incubation of human monocytes on immobilized activating anti-αD antibodies (mAb 169B, 217I) induces release of MCP-1. Wells were coated with anti-αD mAb 169B or 217I, anti-αM, human serum albumin (HSA), or non-immune IgG1 and isolated human monocytes were incubated with these immobilized immunoglobulins and proteins as described in the legend for Table S3. Supernatants were collected at the end of an 8 hr incubation, frozen at −70°, and MCP-1 in the supernatants was later measured by ELISA. The values are in pg/mL. In each experiment, the concentration of MCP-1 was higher in supernatants from monocytes incubated on immobilized anti-αD mAb 169B and 217I than in supernatants from monocytes incubated on control surfaces, although the values varied substantially among individual donors in the 8 experiments. (DOCX) [file pone.0112770.s009.docx]

**Table S5: Incubation of human monocytes on immobilized activating anti-α_D_ antibodies (mAb 169B, 217I) induces release of MCP-1**

| **Experiment** | **HSA** | **IgG1** | **mAb 169B** | **mAb 217I** | **anti-α_M_** |
| --- | --- | --- | --- | --- | --- |
| 1 | 42 | 264 | **597** | **771** | 95 |
| 2 | 28 | 42 | **183** | **95** | 14 |
| 3 | 14 | 55 | **121** | **171** | 14 |
| 4 | 28 | 95 | **350** | **146** | 42 |
| 5 | 41 | 95 | **251** | **158** | 14 |
| 6 | 0 | 46 | **99** | **55** | 24 |
| 7 | 0 | 96 | **597** | **859** | 115 |
| 8 | 0 | 46 | **99** | **55** | 24 |
| Mean | 19 | 92 | **287** | **289** | 43 |
| Range | 0-42 | 42-264 | **99-597** | **55-859** | 14-115 |

Table S5 Legend: Wells were coated with anti-α_D_ mAb 169B or 217I, anti-α_M_, human serum albumin (HSA), or non-immune IgG1 and isolated human monocytes were incubated with these immobilized immunoglobulins and proteins as described in the legend for Table S3. Supernatants were collected at the end of an 8 hr incubation, frozen at -70°, and MCP-1 in the supernatants was later measured by ELISA. The values are in pg/mL. In each experiment, the concentration of MCP-1 was higher in supernatants from monocytes incubated on immobilized anti-α_D_ mAb 169B and 217I than in supernatants from monocytes incubated on control surfaces, although the values varied substantially among individual donors in the 8 experiments.
