# Supplementary material for: Integrin αDβ2 (CD11d/CD18) Is Expressed by Human Circulating and Tissue Myeloid Leukocytes and Mediates Inflammatory Signaling
Source: PLoS One. 2014 Nov 21;9(11):e112770. doi: 10.1371/journal.pone.0112770 (PMC4240710; doi:10.1371/journal.pone.0112770)
Supplement: Table S6 — Expression of IL-1β in human monocytes incubated on immobilized activating anti-αD mAb or control proteins. Isolated human monocytes were incubated on immobilized anti-αD mAb 169B or 217I, anti-αM, human serum albumin (HSA), or non-immune IgG1 as described in Table S3. Supernatants were collected after an 8 hr. incubation and centrifuged as in Table S3. The adherent monocytes were then scraped from the wells, pooled with pellets from centrifugation of the supernatants, and lysed. The supernatants and monocyte lysates were stored separately at -70°C and later assayed for IL-1β by ELISA. The values shown are in pg/mL. (DOCX) [file pone.0112770.s010.docx]

**Table S6: Expression of IL-1β in human monocytes incubated on immobilized activating anti-α_D_ mAb or control proteins**

| **Experiment**  **Lysates** | **HSA** | **IgG1** | **mAb 169B** | **mAb 217I** | **anti-α_M_** |
| --- | --- | --- | --- | --- | --- |
| 1 | 4 | 88 | **192** | **134** | 40 |
| 2 | 29 | 89 | **140** | **130** | 52 |
| 3 | 3 | 53 | **222** | **181** | 40 |
| 4 | 2 | 22 | **31** | **26** | 3 |
| 5 | 6 | 48 | **35** | **40** | 12 |

**Supernatants**

| 1 | 12 | 9 | **118** | **12** | 0 |
| --- | --- | --- | --- | --- | --- |
| 2 | 6 | 9 | **15** | **9** | 6 |
| 3 | 4 | 7 | **62** | **15** | 6 |
| 4 | 9 | 9 | **27** | **12** | 2 |
| 5 | 12 | 9 | **18** | **9** | 12 |

**Total (Lysates +**

**Supernatants)**

| 1 | 16 | 97 | **310** | **146** | 40 |
| --- | --- | --- | --- | --- | --- |
| 2 | 35 | 98 | **155** | **139** | 58 |
| 3 | 7 | 60 | **284** | **196** | 46 |
| 4 | 11 | 31 | **58** | **38** | 5 |
| 5 | 18 | 57 | **53** | **49** | 24 |
| Mean (Total) | 21 | 80 | **172** | **114** | 35 |
| Range | 7-35 | 31-98 | **53-310** | **38-196** | 5-58 |

Table S6 Legend: Isolated human monocytes were incubated on immobilized anti-α_D_ mAb 169B or 217I, anti-α_M_, human serum albumin (HSA), or non-immune IgG1 as described in Table S3. Supernatants were collected after an 8 hr. incubation and centrifuged as in Table S3. The adherent monocytes were then scraped from the wells, pooled with pellets from centrifugation of the supernatants, and lysed. The supernatants and monocyte lysates were stored separately at -70°C and later assayed for IL-1β by ELISA. The values shown are in pg/mL.
